# Effect of sodium bicarbonate on prolonged running performance: A randomized, double-blind, cross-over study

**DOI:** 10.1371/journal.pone.0182158

**Published:** 2017-08-10

**Authors:** Tanja Freis, Anne Hecksteden, Ulf Such, Tim Meyer

**Affiliations:** Institute of Sports and Preventive Medicine, Saarland University, Saarbrücken, Germany; Texas A&M University, UNITED STATES

## Abstract

**Background:**

The ability to sustain intense exercise seems to be partially limited by the body’s capability to counteract decreases in both intra- and extracellular pH. While the influence of an enhanced buffering capacity via sodium bicarbonate (BICA) on short-term, high-intensity exercise performance has been repeatedly investigated, studies on prolonged endurance performances are comparatively rare, especially for running. The aim of the following study was to assess the ergogenic effects of an oral BICA substitution upon exhaustive intensive endurance running performance.

**Method:**

In a double-blind randomized cross-over study, 18 trained runners (VO_2peak_: 61.2 ± 6.4 ml•min^-1^•kg^-1^) performed two exhaustive graded exercise tests and two constant load tests (30 main at 95% individual anaerobic threshold (IAT) followed by 110% IAT until exhaustion) after ingestion of either sodium bicarbonate (BICA) (0.3 g/kg) or placebo (4 g NaCl) diluted in 700 ml of water. Time to exhaustion (TTE) in the constant load test was defined as the main outcome measure. Throughout each test respiratory gas exchange measurements were conducted as well as determinations of heart rate, blood gases and blood lactate concentration.

**Results:**

TTE in the constant load test did not differ significantly between BICA and placebo conditions (BICA: 39.6 ± 5.6 min, placebo: 39.3 ± 5.6 min; p = 0.78). While pH in the placebo test dropped to a slightly acidotic value two minutes after cessation of exercise (7.34 ± 0.05) the value in the BICA trial remained within the normal range (7.41 ± 0.06) (p < 0.001). In contrast, maximum running speed (V_max_) in the exhaustive graded exercise test was significantly higher with BICA (17.4 ± 1.0 km/h) compared to placebo (17.1 ± 1.0 km/h) (p = 0.009). The numerical difference in maximum oxygen consumption (VO_2peak_) failed to reach statistical significance (BICA: 61.2 ± 6.4 ml•min^-1^•kg^-1^, placebo: 59.8 ± 6.4 ml•min^-1^•kg^-1^; p = 0.31). Maximum blood lactate was significantly higher with BICA compared to the corresponding placebo test (BICA: 11.1 ± 2.3 mmol/l, placebo: 8.9 ± 3.0 mmol/l; p < 0.001). At the end of exercise, an acidotic pH value was found in both exhaustive graded exercise tests (p = 0.002). BICA caused gastrointestinal side effects in 15 patients.

**Conclusion:**

Maximal performance was enhanced significantly after BICA administration. The ergogenic effect of BICA in the exhaustive graded exercise test can most likely be attributed to an increased anaerobic glycolysis that is reflected by an accumulation of lactate. However, TTE in prolonged high-intensity running was not improved. Even at the end of exercise no severe metabolic acidosis was found. Metabolic acidification as one of the dominant factors causing muscular fatigue should therefore be reconsidered.

**Trial registration:**

German Clinical Trials Register (DRKS) DRKS00011284.

## Introduction

Exercise-induced acidosis, acid-base balance and related effects on exercise performance have been repeatedly studied over the last decades [1–4]. Even though the aetiology of muscle fatigue is still controversial, an accumulation of hydrogen ions (H^+^) and the concomitant reduction in myoplasmic pH have been assumed as one of the major causes for the onset of exercise-induced fatigue [5–7]. Consequently, the supplementation of alkalizing agents prior to exercise, such as sodium bicarbonate (BICA), is thought to have the potential to neutralize metabolic acidosis as it occurs in heavy exercise by enhancing buffer capacity and thereby delaying the onset of acidosis and fatigue. An increase of extracellular pH may also facilitate the efflux of H^+^ ions from the working muscles [7–10] and thereby contribute to a potential ergogenic effect of BICA. Trials attempting to verify the expected positive effects of sodium bicarbonate on exercise performance focused mainly on high intensity exercise of short duration [2,8,11–14] in which the proportion of glycolytic energy metabolism as well as blood and myocellular lactate concentrations are largest. However, little is known regarding the effects on performance during prolonged intense endurance exercise [15–23]. Although endurance running relies mainly on aerobic energy metabolism, at higher intensities the relevant contribution of anaerobic glycolysis to the total energy production is reflected by an accumulation of lactate and protons [17,19,22]. Hence, improvements in endurance performances with BICA seem plausible. In accordance with this view, six out of nine studies on endurance performances in other sports showed a significant ergogenic effect of BICA compared to placebo [15,16,18,20,21,23]. Despite the popularity of endurance running only two relevant studies [22,23] have been published so far for this exercise mode. Therefore, this study aims to investigate the effects of sodium bicarbonate supplementation on high-intensity endurance performance in runners.

## Methods

The protocol of this trial and supporting CONSORT checklist are available as supporting information; see S1 and S2 Protocols and S1 CONSORT Checklist. Trial protocol provided was approved by the local ethics committee prior to the start of the study. In addition, all relevant data are presented in tabular form; see S1 Data.

### Ethics statement

The present study was carried out in accordance with the declaration of Helsinki. Approval for the study’s procedures was granted by the local ethics committee (Aerztekammer des Saarlandes, Saarbruecken, Germany; approval number: 35/11, March 21^st^ 2011). Reasons for the delayed registering of the study were a low number of subjects and a quick recruitment of participants from a local pool of athletes. The authors confirm that all ongoing and related trials for this intervention are registered.

### Subjects

**Fig 1:**
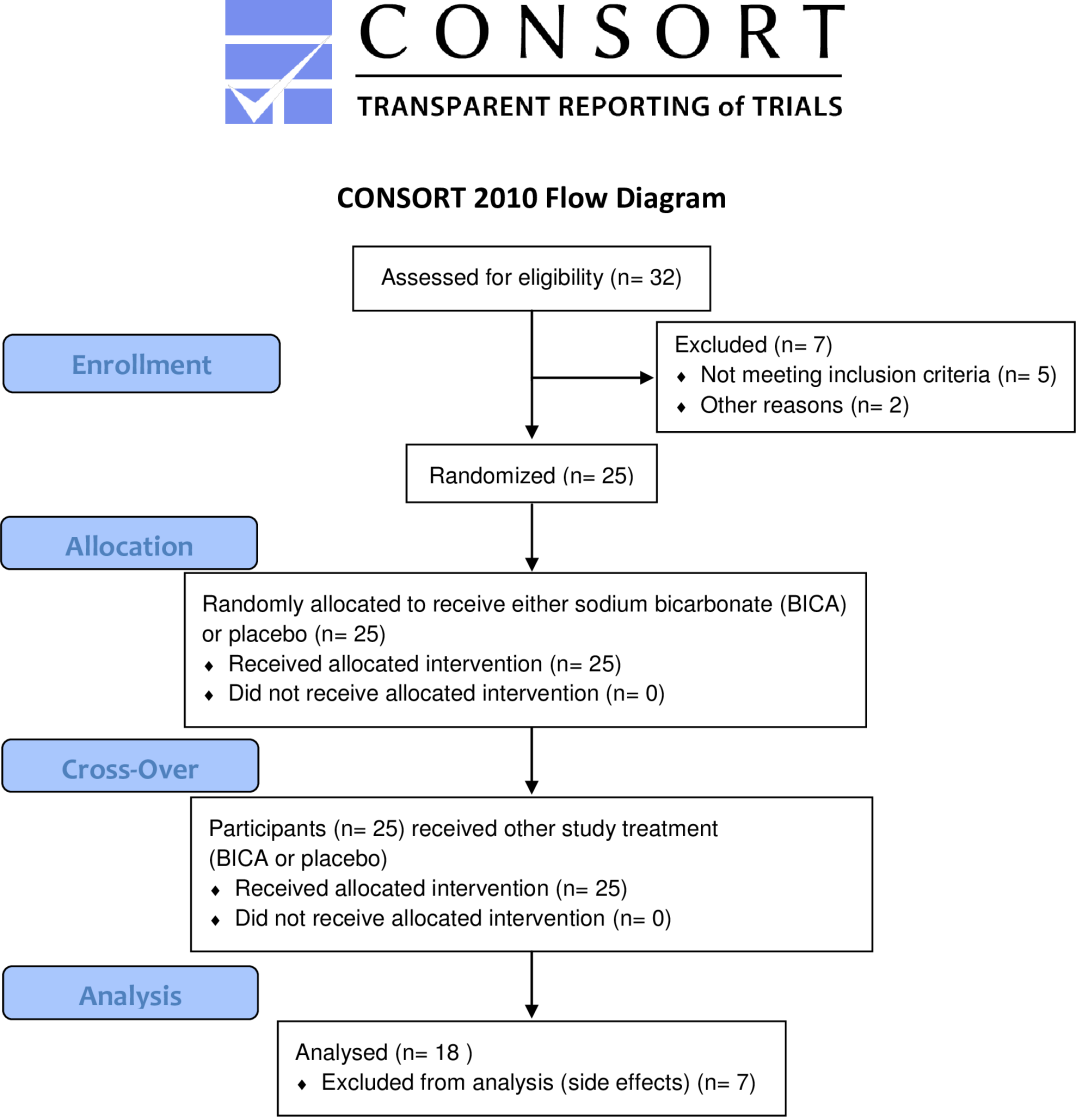
Flow diagram of the study.

Participants were recruited between September 2011 and July 2012. Overall thirty-two healthy endurance athletes (30 males, two females) volunteered to participate in this study. All subjects provided written consent after being informed of possible side effects, study requirements and benefits. Inclusion criteria were: full legal age (18 yrs.) and a maximum running speed of at least 4.5 m/s (4.0 m/s for women) in both graded exercise tests, absence of medical diseases or disorders that would interfere with testing or require medication. From 25 randomized subjects 18 could be included in the final analysis (17 males, 1 female, age: 27.9 ± 9.1 yrs., BMI: 22.4 ± 1.8 kg/m^2^, VO_2max_: 61.2 ± 6.4 ml•min^-1^•kg^-1^). The flow of subjects is depicted in Fig 1. All drop-outs were due to gastrointestinal side effects of BICA.

### General design

**Fig 2:**
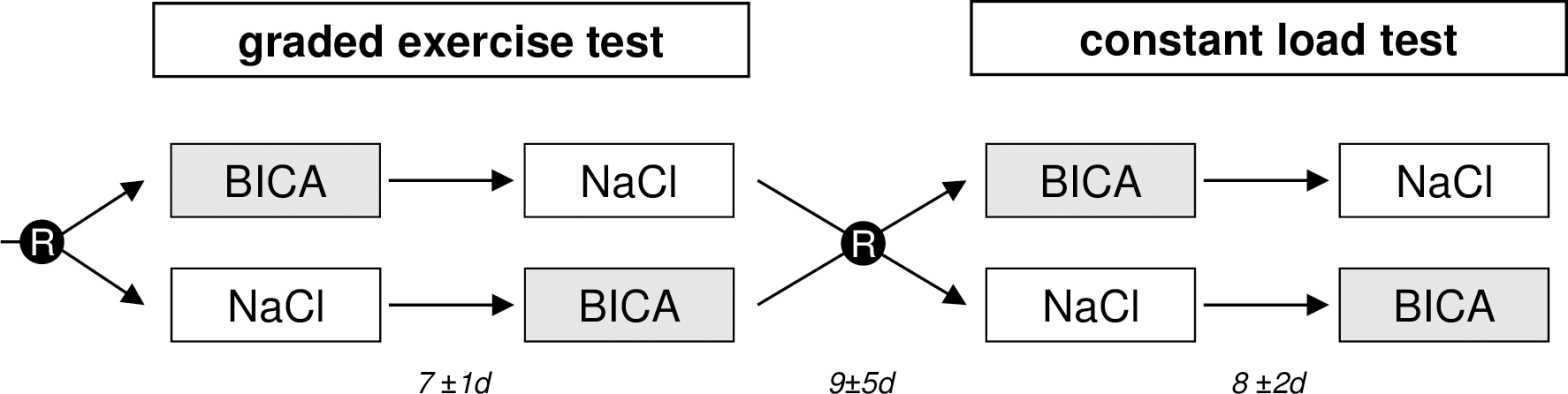
General study design. Subjects were randomly allocated before both the graded exercise and the constant load test. Both test protocols were conducted after ingestion of either 0.3 g/kg BICA or 4 g placebo (NaCl). Time intervals between the tests are presented as means ± SD. R in black circle = randomization.

In a double-blind randomized cross-over trial participants completed four exercise tests to volitional exhaustion: two graded exercise and two constant load tests (see Fig 2). The two treatment solutions, 0.3 g/kg body weight^-1^ sodium bicarbonate (BICA) or 4 g placebo (NaCl) dissolved in 0.7 l of water had to be consumed within a one hour period, starting 1.5 hours prior to the test. Subjects were allowed to drink water ad libitum. Sodium chloride was chosen as the placebo substance because the taste is quite similar to BICA. Prior to the start of the study we tested different sodium chloride dosages and at 4g NaCl none of our test persons could tell the difference to sodium bicarbonate. Subjects in this study also reported similar taste of both the sodium bicarbonate and placebo solution. Subjects reported similar taste of both the sodium bicarbonate and placebo solution. Eligible participants were randomly assigned to sodium bicarbonate or placebo before both the graded exercise and constant load tests by a staff member who had no contact to them. Simple randomization was generated by a random draw of numbers. Treatment allocation was concealed from participants as well as from the researcher responsible for enrolling them and conducting the trials. There was no correction for plasma volume changes during exercise.

### Pretest preparations

The participants were asked to report to the laboratory approximately 2.5 hours before the start of each test. Athletes were instructed to replicate the same dietary intake for three days prior to all tests and avoid strenuous exercise in the 24 hours before each trial. In this context, participants were required to document their training and nutrition of the last 3.5 days. Urine samples were requested shortly before and after ingestion of the test solution and were checked for changes in pH. At the initial test, medical history was obtained and physical examination was performed with additional measurement of blood pressure on both arms as well as body weight and height. Furthermore, to ensure the athletes health a 12-lead ECG was written and a lung function test was performed before the first test.

### Exhaustive graded exercise test

The exhaustive graded exercise tests commenced at 2.5 m/s (2.0 m/s for women). Treadmill speed was increased every three minutes by 0.5 m/s until volitional exhaustion. Vigorous verbal encouragement was provided to each participant towards the end of each trial. The tests were finished when the participants could no longer maintain the speed. Steps were separated by a 30 s interruption for capillary blood sampling. Heart rate (HR) was recorded via ECG monitoring. The exhaustive graded exercise test was used to determine the athlete’s individual anaerobic threshold (IAT) [24]. Since there was no difference between BICA and placebo (p = 0.79) both values could be used for the design of the subsequent constant load tests. IAT was compared as actual speed.

### Constant load test

Initially, a warm-up was performed consisting of a 5 min run at a treadmill speed equal to 60% IAT. As previously published in cyclists [21], the actual test consisted of two parts: First, a 30 min aerobic run starting at 95% IAT followed by exercise at 110% IAT to exhaustion. Heart rate (HR) was recorded every 5 min using a telemetric system (Polar S-series, Kempele, Finnland). Time to exhaustion was calculated as the time run at 110% IAT. Vigorous verbal encouragement was provided to each participant. The tests were finished when the participants could no longer maintain the required speed.

### Blood lactate measurement

Capillary blood samples for the determination of blood lactate concentration (BLa) were collected from the hyperemized ear lobe after each workload in the graded exercise and every 5 minutes in the constant load test. In the graded exercise test, measurements of lactate concentrations were conducted immediately at the end of exercise as well as 1, 3, 5, 7 and 10 min after cessation of exercise. In the constant load test BLa was measured immediately post exercise (post ex).

### Blood gas measurement

Venous blood samples were obtained from venopuncture prior to all trials to measure hemoglobin and potassium concentration. The hemoglobin concentration obtained was needed to calculate blood gases. Blood gas analyses included pH, partial pressure of carbon dioxide (pCO_2_), partial pressure of oxygen (pO_2_), oxygen saturation (SO_2_), standard bicarbonate (SBi), and base excess (BE) (ABL5 blood gas analyzer, Radiometer, Willich, Germany). Blood gases were determined shortly before (pre ing) and after the ingestion of the test solution (post ing) and immediately before the test. During the course of the constant load test, blood gas measurements were conducted every five minutes. Blood gas analysis was performed in the 2^nd^ min of recovery (post ex) for both testing protocols.

### Respiratory gas exchange analysis

During all tests, respiratory gases were constantly collected and analyzed via the Metalyzer or MetaMax II metabolic test system (Cortex Biophysik, Leipzig, Germany) using the same system for all trials of a given subject. Subjects breathed through a Hans-Rudolph-facemask that was selected prior to the first exhaustive graded exercise test and remained the same during the course of the study. According to the manufacturer's instructions, a calibration was performed before each use.

### Outcome measures

Time to exhaustion in the constant load test was defined as the main outcome measure. Secondary outcome measures included parameters of maximal performance in the exhaustive graded exercise test such as maximum running speed and VO_2peak_ as well as parameters most likely related to a potential performance enhancing effect of BICA and muscle fatigue (pH, sodium bicarbonate concentration, base excess and blood lactate).

### Statistical analysis

All statistical analyses were completed using Statistica (Version 8, StatSoft Inc., Tulsa, USA), with significance set at p < 0.05 for the α-error. Data was analyzed using parametric tests following confirmation of a normal distribution via Shapiro-Wilks-W-test and are presented as mean ± standard deviation (SD). A one-factor analysis of variance for repeated measures (ANOVA, factor: condition) was employed to test for significant differences between the sodium bicarbonate and the placebo trial. When the ANOVA revealed a significant effect for one of the factors or their interaction, the Scheffé post-hoc test was applied.

Sample size was calculated with the program G*Power (version 3.1.5) using the following parameters [25]: Statistical test: repeated measures ANOVA between factors, effect size to be detected 0.6, alpha error probability 0.05, 1-beta error probability 0.80; correlation among repeated measures 0.6. The resulting target sample size was 19. Allowing for a dropout rate of 25%. Due to a higher rate of side effects the target sample size was missed by one subject. Achieved power is 0.78.

## Results

### Performance

Time to exhaustion in the constant load test did not differ between conditions (BICA: 39.6 ± 5.6 min, placebo: 39.3 ± 5.6 min; p = 0.78). By contrast, maximum running speed (V_max_) in exhaustive graded exercise test was significantly higher (p = 0.009) in the BICA (17.4 ± 1.0 km/h) as compared to the placebo trial (17.1 ± 1.0 km/h). No significant difference in maximum running speed (V_max_) was detected when comparing the participants that started with placebo to those who started with BICA and vice versa. Peak oxygen uptake (VO_2peak_) and individual anaerobic threshold (IAT) did not differ significantly between conditions (VO_2peak_: p = 0.31, IAT: p = 0.79).

### Blood gas analysis

**Fig 3:**
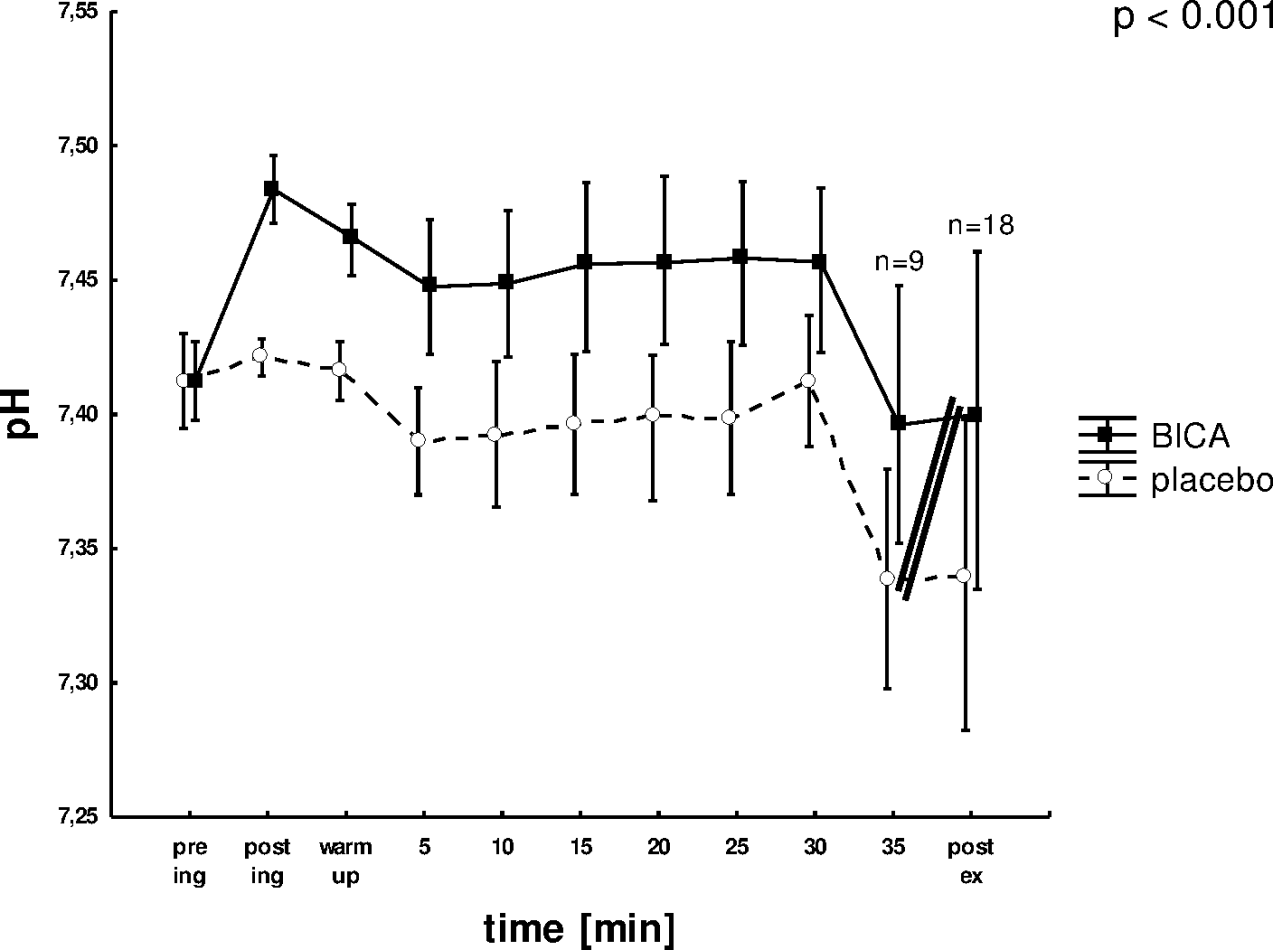
pH prior (pre ing), after ingestion (post ing), during the constant load test and post exercise (post ex) for BICA (closed squares) and placebo (open circles). Double bar indicates end of exercise. Data are expressed as means ± SD. p = interaction between condition and time.

Pre-ingestion all blood gas parameters were similar between conditions and within normal resting levels for all tests. Tables 1 and 2 summarize selected blood gas values during the constant load and exhaustive graded exercise tests, respectively. Fig 3 illustrates the changes in pH with time in the constant load test. In the constant load tests, blood pH was found to be significantly higher with BICA compared to placebo over the entire test period (p<0.001). While the pH in the placebo test dropped to a slightly acidotic value two minutes after the cessation of exercise (post ex) the value in the BICA trial remained within the normal range (7.36–7.45). The mean SBi concentration and BE values remained significantly higher at all sampling times following ingestion (p<0.001). The mean SBi concentration dropped from post ingest to the 2^nd^ min of recovery (post ex) to a similar extent (p = 0.01). In the exhaustive graded exercise test a metabolic acidosis was found at the sampling time post exercise in both the placebo and the BICA test. In the course of the exhaustive graded exercise test, the drop in SBi concentration from post ingest to post exercise was more pronounced with BICA as compared to placebo (p<0.001).

**Table 1:** Blood gas parameters during constant load test.

	placebo (n = 18)	BICA (n = 18)	p-value
**pH**			
post ing	7.42 ± 0.02	7.48 ± 0.02	<0.001
30 min	7.41 ± 0.03	7.46 ± 0.03	<0.001
post ex	7.34 ± 0.05	7.40 ± 0.07	<0.001
**SBi** [mmol/l]			
post ing	24.9 ± 1.7	31.1 ± 2.2	<0.001
30 min	22.6 ± 2.5	27.5 ± 3.0	<0.001
post ex	17.7 ± 3.1	21.9 ± 3.7	<0.001
**BE** [mmol/l]			
post ing	1.0 ± 1.7	7.2 ± 2.2	<0.001
30 min	-1.4 ± 2.6	3.8 ± 3.1	<0.001
post ex	-7.2 ± 3.6	-2.2 ± 4.4	<0.001

pH value, sodium bicarbonate concentration (SBi) and base excess (BE) were conducted at rest (post ing), after 30 minutes of running and two minutes post exercise (post ex). Data are presented as means ± SD. Significance was tested using one-factorial repeated measures analysis of variance (factor: condition; post-hoc: Scheffé test).

**Table 2:** Blood gas parameters during exhaustive graded exercise test.

	placebo (n = 18)	BICA (n = 18)	p-value
**pH**			
post ing	7.42 ± 0.01	7.49 ± 0.03	<0.001
post ex	7.27 ± 0.05	7.30 ± 0.07	0.002
**SBi** [mmol/l]			
post ing	24.9 ± 1.4	31.8 ± 3.0	<0.001
post ex	15.9 ± 3.9	17.7 ± 3.5	<0.001
**BE** [mmol/l]			
post ing	1.2 ± 2.0	8.2 ± 2.8	<0.001
post ex	-10.2 ± 3.2	-7.4 ± 4.4	<0.001

pH value, sodium bicarbonate concentration (SBi) and base excess (BE) were conducted at rest (post ing) and two minutes post exercise (post ex). Data are presented as means ± SD: Significance was tested using one-factorial repeated measures analysis of variance (factor: condition; post-hoc: Scheffé test).

### Blood lactate concentration

No significant differences were observed between the BICA and placebo conditions for baseline levels of blood lactate. Blood lactate concentrations during and after exercise were significantly higher after BICA supplementation when compared to placebo in the constant load test (p<0.001). Similar results were found in the exhaustive graded exercise test measured from post ingest throughout the entire test (p<0.001).

### Respiratory gas exchange analysis

Although placebo appeared to elicit numerically higher levels for the mean ventilation (VE) throughout the test (except pre ingest), the difference between conditions failed to reach statistical significance (exhaustive graded exercise test p = 0.06; constant load test p = 0.19). In addition, neither oxygen uptake (VO_2_) nor carbon dioxide output (VCO_2_) achieved statistical significant differences between the courses during both test types (exhaustive graded exercise test VCO_2_: p = 0.06, VO_2_: p = 0.98; constant load test VCO_2_: p = 0.89, VO_2_: p = 0.74).

### Side effects

Overall, 15 patients suffered from side effects during the course of the study and seven of them reported the respective complaints as the reason of exercise cessation. The side effects included stomach ache (n = 6), diarrhea (n = 5), nausea/vomiting (n = 2) and dizziness (n = 2). Side effects only emerged after sodium bicarbonate supplementation, most often during the constant load tests (n = 11). Three athletes reported adverse effects in both the graded exercise and constant load test with BICA. One athlete suffered from diarrhea after consuming BICA and for this reason could not commence the respective constant load test. The participant agreed to repeat the test one week later at the same time and no gastrointestinal disturbances occurred. The data obtained during this additional test were not included in the main analysis.

## Discussion

Oral ingestion of BICA did not elicit a significant improvement in time to exhaustion in the constant load test. By contrast, maximum running speed (V_max_) in the exhaustive graded exercise test was significantly higher with BICA compared to placebo. Beyond these measures of physical performance, the absence of a metabolic acidosis at the end of exercise in the constant load test with BICA was one of the major findings in the present study. Moreover, it is of note that almost 50% of the subjects suffered from side effects after ingestion of BICA with a peak occurrence at 110% IAT in the constant load test. No gastrointestinal disturbances were reported after intake of the placebo solution. In an applied setting, side effects could well counteract BICA’s potential performance enhancing effect. However, they do not explain the lack of improvement in TTE in this trial because only participants who did not experience any side effects were included in the main analysis.

As mentioned above, TTE in prolonged high-intensity running was not improved via intake of BICA. By contrast, Georg et al. [23] investigated the effects of 0.2 g/kg BICA on running performance corresponding to a blood lactate concentration of 4 mM in seven male athletes. The authors report an improvement in time to exhaustion (TTE) by 17% with BICA compared to the placebo group (p<0.01). The study published by Potteiger et al. [22] used a test protocol similar to this trial and showed a numerical increase of endurance performance. Nevertheless, difference failed to reach statistical significance. Apart from running, Egger et al. [21] observed an improved cycling time to exhaustion (p<0.05) with BICA compared to placebo using an identical design in cycling compared to the present trial. In the endurance cycling test by McNaughton et al. [18] subjects were instructed to perform as much work as possible within 60 min resulting in a 14% increase in performance in the BICA compared to the placebo group. However, they doubted whether their subjects exercised above their IAT for a prolonged period of time. The study conducted by Stephens et al. [19] observed no difference in time to exhaustion after one hour of cycling consisting of 30 min at 77% VO_2peak_ followed by completing as much work as possible in another 30 min in a sample of six subjects.

At the end of exercise in both constant load tests no severe metabolic acidosis could be observed. Similarly, Potteiger et al. [22] and McNaughton et al. [18], who also examined the effects of BICA on endurance performances found no significant acidosis post exercise, either, with pH values between 7.35 to 7.45. Likewise, Egger et al. [21] observed no critical drop of the pH value with BICA and only a slightly acidotic value with placebo. It can be assumed that under these study conditions despite exercising above IAT the body’s buffer potential had not yet been completely depleted. Thus it is possible that the physical exhaustion in the BICA constant load trial was not due to an acidification. Following ingestion of BICA significantly lower intramuscular hydrogen ion concentrations (p<0.05) at 30 min and at the end of exercise were found in the study conducted by Stephens et al. [19]. This indicates a delayed pH drop with longer maintenance of energy supply via anaerobic glycolysis in the muscle. Nevertheless, BICA supplementation failed to enhance performance which suggests that the effects of a proton accumulation and the associated metabolic acidosis upon muscle fatigue are not as drastic as previously thought.

After intravenous infusion of both BICA and NaCl cycling performance (30 min at 80% VO_2max_) was significantly improved compared to the control condition with no infusion [17]. However, the research group questioned whether the enhanced performance could be attributed to the prevention of acidosis since alkalosis prior to exercise was only achieved after ingestion of BICA. It is possible that the sodium content of the test solution might influence performance. Hence, NaCl as a placebo substance might itself have an ergogenic effect. By supplementing equal amounts of sodium no significant difference in time to exhaustion between the BICA and NaCl trial in repeated 1-min intervals at 95% VO_2max_ followed by 1 min recovery at 60 W until exhaustion on the cycle ergometer was found [26]. Driller et al. [27] also compared sodium bicarbonate and NaCl with equimolar sodium content to a physically inert placebo (dextrose monohydrate) in eight trained cyclists. They found a significant performance enhancement of sodium bicarbonate when compared to both NaCl and a placebo substance. Nevertheless there was no statistically significant difference in mean power between the NaCl and placebo. Kozak-Collins et al. [26] and Mitchel et al. [17] did not consider the maintenance of the acid-base equilibrium as the mechanism of action of sodium bicarbonate but rather plasma volume expansion. In the process of absorbing sodium ingested with BICA or NaCl from the intestine into the blood, water shifts in response to the osmotic gradient established by sodium resulting in an increase of extracellular fluid. This in turn might result in a better perfusion and oxygen supply of the working muscles. If the sodium content of the solutions improved muscle perfusion and therefore the muscular oxygenation, it is conceivable that the pulmonary oxygen uptake (VO_2_) could have been increased as well. Since the osmotic concentration of the BICA solution was 3.5 times as high as the placebo solution it can be assumed that the VO_2_ should have been higher in the BICA trial, too. However based on the data, this could not be confirmed as the oxygen uptake did not significantly differ among the two conditions (p = 0.77). Yet, we cannot rule out that the higher blood volume did alter cardiovascular strain.

As indicated by our study results the values for maximum lactate were higher in the exhaustive graded exercise test which therefore seems to be more dependent on anaerobic glycolysis than the constant load test. It can also be assumed that in the exhaustive graded exercise test more protons were neutralized as suggested by the greater drop of the SBi concentration. This in turn might attenuate the drop in myoplasmic pH for a longer period of time entailing that glycolytic enzymes, in particular the glycogen phosphorylase and pyruvate dehydrogenase [7,10,28] were inhibited to a lesser extent allowing a longer-running anaerobic glycolysis and therefore an enhanced performance. Towards the end of exercise the accumulating protons eventually led to a restriction of glycolysis and finally to the cessation of exercise as a result of the emerging metabolic acidosis. Overall, the performance enhancing effect of BICA in the exhaustive graded exercise test was consistent with other studies that suggested an improved effectiveness of BICA at maximum load exercises [7,29].

Carr et al. [30] found the highest rate of side effects 90 min after BICA ingestion which coincides with the increased occurrence of gastrointestinal distress at 110% IAT. With an average weight of about 70 kg, the subjects consumed approximately 5.5 g of sodium in the BICA trial. To compensate for this hypertonic BICA solution, large amounts of fluid were likely drawn from the plasma into the gastrointestinal tract provoking stomach pain and diarrhea [31]. The study conducted by Van Montfoort et al. [14] describes only considerable gastrointestinal disturbances after NaCl supplementation when the osmolarity was equal to the BICA solution. This suggests that the amount of salt can be regarded as the major cause of side effects. Applied to the present study, this conclusion can be considered plausible because the placebo solution contained a lower amount of sodium (1.6 g) provoking no side effects. In the study by Egger et al. [21] on the cycle ergometer no participant reported gastrointestinal discomfort despite the same dosage and administration time of BICA. In addition to the hyperosmolarity, up and down movements of organs during running might, thus, aggravate gastrointestinal side effects [32] since mechanical bouncing of the guts is almost twice as high during running as it is in cycling [33]. It is possible that an alternative loading protocol might reduce gastrointestinal distress. Another administering method for sodium bicarbonate is in form of capsules. Capsules seem to minimize gastrointestinal side effects [30] without altering performance in comparison to a solution [34]. A disadvantage is that a large number of capsules have to be ingested.

Driller et al (2012) [35] examined the effects of serial BICA loading (a split dose of 0.4 g/kg BICA taken over a period of three days before exercise) compared to acute loading (0.3 g/kg BICA 90 minutes before test) and placebo (microcrystalline cellulose) in eight well-trained cyclists. Both BICA protocols produced a significantly higher average power in a 4-minute-cycling performance but only in the acute setting three incidences of gastrointestinal side effects (mild bloating and nausea) were reported. A serial loading protocol avoids the intake of BICA on race day and could therefore reduce gastrointestinal distress [36]. The study conducted by Edge et al. (2006) [37] examined the effects of a chronic intake of BICA during interval training and found greater improvements in lactate threshold (p = 0.05) and time to fatigue (p = 0.05) compared to placebo (NaCl). The question rises whether athletes could train harder or could achieve the same level of improvement in a shorter time or with less effort. However, a disadvantage of studies using a chronic protocol might be a lack of compliance by the study participants. Health problems should also be taken into account because chronic intake might increase the risk of cardiac dysrhythmia due to changes in blood potassium concentration and might cause urolithiasis due to the alkalization of urine and the sodium content [38].

After initial incompatibility of BICA, no more gastrointestinal distress occurred in one subject in repetition of the test under the same conditions one week later. This indicates that the tolerance of BICA might not only be individually different, but might be dependent on the current condition of the participants, too.

## Limitations

When interpreting our results, it has to be kept in mind that tests under laboratory conditions, especially with treadmill stops for the conduction of blood gases and lactate, do not entirely correspond to races that participants usually take part in. For example individual pacing strategies had not been taken into account since participants could not alter the velocity on the treadmill. Hence, a possible ergogenic effect of BICA might have been disguised. Yet it is also conceivable that BICA might be less effective under race conditions since this particular study design was chosen in order to maximize the potential beneficial effects of BICA. Therefore it is difficult to extrapolate results obtained in this study to competitive sport. Further trials are needed to validate BICA’s effectiveness under race situations.

Until now, administration protocols on BICA are inconsistent in terms of dose, ingestion period and coingestion with fluid. We choose a dosage of 0.3 g/kg that showed the greatest effect on performance [31,39]. Larger amounts seemed to increase gastrointestinal disturbances without enhancing performance [39]. Nevertheless, in many cases the intake of BICA caused side effects that mainly affected the gastrointestinal tract. A possibility to avoid gastrointestinal disturbances might be the use of a different loading protocol, especially serial loading [35,36] or the intake of sodium bicarbonate in form of capsules [30]. There are still many unanswered questions about the pathophysiology and quantity of side effects caused by BICA which entails a need for further research.

Changes in the concentrations of female reproductive hormones during the course of the menstrual cycle may be associated with changes physiological variables relevant for physical performance. However, the results of trials that actually investigated differences in physical performance between cycle phases are inconclusive–indicating slightly better performance during luteal phase [40], follicular phase [41] or no difference [42]. Based on these reports, a relevant impact of the menstrual cycle seems improbably. It is unlikely that there was any control for menstrual phase since only one woman was included in the final analysis

## Conclusion

In conclusion, the ingestion of BICA showed no ergogenic effect on prolonged high-intensity running but enhanced maximal performance during graded testing. In the constant load test conducted with BICA the pH value remained within the normal range post exercise. Metabolic acidification as one of the dominant factors causing muscular fatigue should therefore be reconsidered. The relationship between pH value and muscle fatigue remains a matter of debate. In many cases, the administration of sodium bicarbonate prior to exercise is accompanied by gastrointestinal side effects such as stomach ache, diarrhea and nausea. Furthermore, they seemed to occur with a high degree of intraindividual differences.

## Supporting information

S1 CONSORT Checklist (DOCX)

S1 Protocol Study protocol. The original study protocol has been carefully translated into English language.
(DOCX)

S2 Protocol Study protocol in original language (German). (DOCX)

S1 Data Tabular presentation of all relevant data. (DOCX)
