# Supplementary material for: Effect of sodium bicarbonate on prolonged running performance: A randomized, double-blind, cross-over study
Source: PLoS One. 2017 Aug 10;12(8):e0182158. doi: 10.1371/journal.pone.0182158 (PMC5552294; doi:10.1371/journal.pone.0182158)
Supplement: S1 Protocol — The original study protocol has been carefully translated into English language. (DOCX) [file pone.0182158.s002.docx]

***Trial study protocol approved by the local ethics committee***

***(Aerztekammer des Saarlandes, Saarbruecken, Germany)***

***Effects of* sodium *bicarbonate on performance in endurance athletes***

***(runners)***

Purpose:

Does buffering of metabolic acids via sodium bicarbonate lead to performance

improvements in an exhaustive graded exercise and constant load test?

Subjects:

Well-trained triathletes, middle and long distance runners; age > 18 years

General design:

Initially, participants receive a medical history followed by a physical examination and anthropometric measurements, an ECG, a pulmonary function test with measurement of inspiratory pressures (PImax/P0.1) and a venous blood sample (to exclude kidney diseases and mineral imbalances).

At an interval of one week two exhaustive treadmill ergometries (exhaustive graded exercise test) with respiratory gas exchange measurements and determination of the individual anaerobic threshold (IAT) will be conducted. In a double-blind, randomised manner subjects ingest a sodium bicarbonate solution (0.3g/kg solved in 0.7 liter water, consumed within 60 min) or an equal volume of a placebo solution two hours prior to each test.

One or two weeks later, participants are asked to perform two running ergometries constant load test) consisting of a workload equal to 95% IAT for 30 min followed by 110% IAT until exhaustion. For this purpose the IAT from the exhaustive graded exercise test with placebo is being used. Again, subjects ingest a solution of sodium bicarbonate or placebo prior to testing. Blood gas analyses from the hyperemized ear lobe were conducted prior to ingestion of the test substances, before testing, every 5 min during exercise and post exercise. Additionally, blood lactate is analyzed every 5 min during exercise, at exhaustion and 1, 3, 5 min post exercise.

During the test period subjects are not allowed to take part in a competition or other season highlights. On the day before testing subjects are instructed to exercise at low intensity, if necessary, and to eat sufficiently. On the examination day, a sufficient dietary intake should be followed up to three hours before testing.

Exclusion criteria:

Acid-base or electrolyte imbalances; cardiovascular diseases or doubts about full physical fitness; maximal performance < 16.2km/h (4.5 m/s).

Hypotheses:

Sodium bicarbonate leads to

- in the exhaustive graded exercise test

- no alteration of the individual anaerobic threshold (IAT)
- an increase of maximal performance
- an elevated maximal lactate with a drop in pH-value.

- in the constant load test:

- prolonged running time and accordingly later onset of fatigue

Purposes:

Effects of sodium bicarbonate on maximal performance, IAT, exercise lactate curves and peak oxygen uptake in the exhaustive graded exercise test, performance in the constant load test.

1. Formalities:

1.1 **Designation of operation**

Effects of sodium bicarbonate on performance in endurance athletes

1.2 **Name of the responsible project leader and the involved physicians:**

Prof. Dr. med. Tim Meyer, Dr. med. Ulf Such

1.3 **Type and number of inspection authorities and names of physicians in the case of multicenter studies**

Reserved.

1.4 **Name and address of the sponsor**

Reserved.

1.5 **Has there ever been such a request to another ethics committee?**

No.

1.5.1 **To which?**

Reserved.

1.5.2 **Submission of the vote including the restrictions of this ethics committee including the potentially exchanged correspondence.**

Reserved.

2. Description and scientific justification of the project

2.1 **Explanation of the study objective**

Numerous studies have shown that the intake of sodium bicarbonate can improve performance mainly in anaerobic exercise lasting 1 to 7 minutes [1,2]. Even though less research has been done, endurance performance does not seem to be influenced [3]. This study aims to examine whether anaerobic endurance performance (treadmill) can be improved after exercising at an intensity just below the individual anaerobic threshold by the ingestion of sodium bicarbonate prior to exercise. It is conceivable that buffering capacity will already be depleted by the time anaerobic exercise starts. By analysing acid-base balance during exercise we will be able to document accurate courses of pH and buffering capacity.

2.2 **Description of the current state of knowledge [2], [4-6]**

There is consensus that muscular fatigue at high-intensity exercise is at least in part due to the decrease of intramuscular pH. Anaerobic energy supply leads to an accumulation of acids (especially lactic acid) which dissociates into lactate ions and hydrogen ions thereby decreasing the pH of muscle and blood. The activity of intracellular enzymes (e.g. the key enzyme of glycolysis: phosphofructokinase) is pH-dependent and decreases with increasing acidosis. Hydrogencarbonate is an important extracellular buffer. A higher extracellular buffering capacity leads to an accelerated efflux of accumulating intracellular hydrogen ions, so that pH can be kept constant at similar accumulation of acids and accordingly similar metabolic activity.

2.3 **Results of the pharmacological-toxicological pre-examination (laboratory tests and animal experiments)**

Reserved.

2.4 **Submission of the entire test plan**

Subjects:

well-trained triathletes, middle or long distance runners; age > 18 years

General design:

Initially, participants receive a medical history followed by a physical examination and anthropometric measurements, an ECG, a pulmonary function test with measurement of inspiratory pressures (PImax/P0.1) and a venous blood sample (to exclude kidney diseases and mineral imbalances).

At an interval of one week two exhaustive treadmill ergometries (exhaustive graded exercise test) with respiratory gas exchange measurements and determination of the individual anaerobic threshold (IAT) will be conducted. In a double-blind, randomised manner subjects ingest a sodium bicarbonate solution (0.3g/kg solved in 0.7 liter water, consumed within 60 min) or an equal volume of a placebo solution two hours prior to each test.

One or two weeks later, participants are asked to perform two running ergometries constant load test) consisting of a workload equal to 95% IAT for 30 min followed by 110% IAT until exhaustion. For this purpose the IAT from the exhaustive graded exercise test with placebo is being used. Again, subjects ingest a solution of sodium bicarbonate or placebo prior to testing. Blood gas analyses from the hyperemized ear lobe were conducted prior to ingestion of the test substances, before testing, every 5 min during exercise and post exercise. Additionally, blood lactate was analyzed post exercise.

2.5 **Envisaged overall duration of examination**

6 months.

2.6 **Justification for the need of studies on humans**

An animal model which is predictive to human outcomes is not available for this scientific question.

2.6.1 **Study on healthy subjects?**

Yes.

2.6.2 **Study on patients?**

No.

2.6.3. **Inclusion criteria**

1.) Age of majority

2.)Good performance capacity with maximal performance of at least <16.2km/h (4.5 m/s) in runners in the exhaustive graded exercise test.

2.6.4 **Exclusion criteria**

1.) Acid-base or electrolyte imbalances

2.) Cardiovascular diseases or doubts about full physical fitness

3.) Current intake of medication

2.6.5 **Intermediate exclusion criteria**

Acute injuries or diseases.

2.6.6 **Concurrent medication**

Reserved.

2.6.7 **Indication of side effects**

Sodium bicarbonate may decrease the blood potassium level. By means of blood samples a preexisting hypocalcaemia can be excluded. Sodium bicarbonate is freely marketed, non-pharmacy-restricted and completely unproblematic concerning doping guidelines.

2.6.8 **Statement to possible risks and side effects (including the not yet**

**described)**

Though no complications are expected complete emergency equipment is available.

2.6.9 **Discontinuation criteria**

1.) The subject’s decision to withdraw from the study

2.) (gastrointestinal) intolerance of sodium bicarbonate

3.) Health problems, which manifest in the course of the study

2.7.0 **Designation of advisory and control commission**

Reserved.

3 References:

1. Lindermann JK GK (1994) The effects of sodium bicarbonate ingestion on exercise performance. Sports Med 18: 75-80.

2. McNaughton LR, Siegler J, Midgley A (2008) Ergogenic effects of sodium

bicarbonate. Curr Sports Med Rep 7: 230-236.

3. George KP, MacLaren DP (1988) The effect of induced alkalosis and acidosis on endurance running at an intensity corresponding to 4 mM blood lactate. Ergonomics 31: 1639-1645.

4. Lindermann JK FT (1991) Sodium bicarbonate ingestion and exercise

performance. An update. Sports Med 11: 71–77.

5. Kemp G, Boning D, Beneke R, Maassen N (2006) Explaining pH change in

exercising muscle: lactic acid, proton consumption, and buffering vs. strong ion difference. Am J Physiol Regul Integr Comp Physiol 291: R235-237;

author reply R238-239.

6. Zinner C, Wahl P, Achtzehn S, Sperlich B, Mester J (2011) Effects of bicarbonate ingestion and high intensity exercise on lactate and H(+)-ion distribution in different blood compartments. Eur J Appl Physiol 111: 1641-1648.
