# Supplementary material for: Effect of sodium bicarbonate on prolonged running performance: A randomized, double-blind, cross-over study
Source: PLoS One. 2017 Aug 10;12(8):e0182158. doi: 10.1371/journal.pone.0182158 (PMC5552294; doi:10.1371/journal.pone.0182158)
Supplement: S1 Data — (DOCX) [file pone.0182158.s004.docx]

| **Baseline characteristics** | | | | | | |
| --- | --- | --- | --- | --- | --- | --- |
| **n** | **gender**  male=1.  female=2 | **weight**  [kg] | **height**  [m] | **BMI**  [kg/m^2^] | **age**  [y] | **VO_2peak_**  [ml•min^-1^•kg^-1^] |
| 1 | 1 | 81 | 1.81 | 24.7 | 27 | 60.4 |
| 2 | 1 | 81 | 1.84 | 23.9 | 36 | 70.3 |
| 3 | 1 | 80 | 1.85 | 23.4 | 18 | 62.4 |
| 4 | 1 | 84 | 1.91 | 23.0 | 23 | 59.8 |
| 5 | 1 | 76 | 1.83 | 22.7 | 20 | 63.9 |
| 6 | 1 | 80 | 1.79 | 25.0 | 40 | 57.0 |
| 7 | 1 | 74 | 1.8 | 22.8 | 52 | 54.2 |
| 8 | 1 | 76 | 1.84 | 22.4 | 30 | 57.9 |
| 9 | 1 | 63 | 1.75 | 20.6 | 26 | 61.7 |
| 10 | 1 | 75 | 1.73 | 25.1 | 41 | 59.2 |
| 11 | 1 | 69 | 1.83 | 20.6 | 26 | 64.9 |
| 12 | 1 | 66 | 1.72 | 22.3 | 24 | 58.0 |
| 13 | 1 | 82 | 1.86 | 23.7 | 23 | 52.0 |
| 14 | 1 | 73 | 1.81 | 22.3 | 27 | 68.4 |
| 15 | 1 | 70 | 1.92 | 19.0 | 30 | 74.1 |
| 16 | 1 | 63 | 1.72 | 21.3 | 19 | 53.8 |
| 17 | 1 | 68 | 1.78 | 21.5 | 21 | 70.0 |
| 18 | 2 | 59 | 1.73 | 19.7 | 19 | 53.1 |

**exhaustive graded exercise (placebo)**

| **n** | **pH pre-ing** | **pH post-ing** | **pH post-ex** | **SBi pre-ing**  [mmol•l^-1^] | **SBi post-ing**  [mmol•l^-1^] | **SBi post-ex**  [mmol•l^-1^] | **BE pre-ing**  [mmol•l^-1^] | **BE post-ing**  [mmol•l^-1^] | **BE post-ex**  [mmol•l^-1^] |
| --- | --- | --- | --- | --- | --- | --- | --- | --- | --- |
| 1 | 7.42 | 7.42 | 7.14 | 27 | 28 | 12 | 4 | 3 | -15 |
| 2 | 7.40 | 7.44 | 7.36 | 24 | 23 | 18 | -1 | -1 | -6 |
| 3 | 7.40 | 7.40 | 7.30 | 26 | 25 | 15 | 2 | 1 | -10 |
| 4 | 7.40 | 7.41 | 7.30 | 26 | 25 | 17 | 2 | 1 | -9 |
| 5 | 7.41 | 7.45 | 7.25 | 28 | 25 | 14 | 3 | 2 | -12 |
| 6 | 7.41 | 7.39 | 7.28 | 25 | 23 | 13 | 1 | -1 | -12 |
| 7 | 7.41 | 7.41 | 7.26 | 25 | 25 | 17 | 1 | 1 | -9 |
| 8 | 7.41 | 7.40 | 7.33 | 27 | 26 | 20 | 3 | 2 | -5 |
| 9 | 7.42 | 7.42 | 7.30 | 26 | 26 | 16 | 2 | 2 | -9 |
| 10 | 7.43 | 7.41 | 7.27 | 25 | 25 | 17 | 1 | 1 | -9 |
| 11 | 7.41 | 7.40 | 7.31 | 26 | 25 | 16 | 2 | 0 | -9 |
| 12 | 7.41 | 7.42 | 7.25 | 30 | 25 | 15 | 1 | 7 | -11 |
| 13 | 7.42 | 7.42 | 7.29 | 26 | 25 | 15 | 2 | 2 | -10 |
| 14 | 7.43 | 7.42 | 7.28 | 26 | 26 | 14 | 2 | 2 | -12 |
| 15 | 7.44 | 7.42 | 7.21 | 24 | 24 | 16 | 4 | 2 | -11 |
| 16 | 7.42 | 7.43 | 7.29 | 23 | 22 | 16 | -1 | -2 | -9 |
| 17 | 7.43 | 7.42 | 7.30 | 26 | 25 | 18 | 2 | 1 | -7 |
| 18 | 7.42 | 7.42 | 7.19 | 23 | 23 | 9 | -1 | -1 | -19 |
| *pre-ing = pre ingestion; post-ing = post ingestion; post-ex = 2 minutes post exercise;*  *SBi = sodium bicarbonate concentration; BE = base excess* | | | | | | | | | |

**exhaustive graded exercise (placebo)**

| **n** | **pCO_2_ pre-ing**  [mmHg] | **pCO_2_ post-ing**  [mmHg] | **pCO_2_ post-ex**  [mmHg] | **pO_2_ pre-ing**  [mmHg] | **pO_2_ post-ing**  [mmHg] | **pO_2_ post-ex**  [mmHg] | **SO_2_ pre-ing**  [%] | **SO_2_ post-ing**  [%] | **SO_2_ post-ex**  [%] |
| --- | --- | --- | --- | --- | --- | --- | --- | --- | --- |
| 1 | 43 | 44 | 36 | 86 | 76 | 86 | 95 | 93 | 94 |
| 2 | 39 | 34 | 33 | 75 | 123 | 96 | 95 | 99 | 97 |
| 3 | 44 | 40 | 32 | 72 | 84 | 99 | 94 | 96 | 97 |
| 4 | 43 | 40 | 34 | 84 | 93 | 87 | 96 | 97 | 96 |
| 5 | 45 | 37 | 34 | 75 | 84 | 87 | 95 | 97 | 95 |
| 6 | 40 | 38 | 29 | 92 | 88 | 98 | 97 | 97 | 97 |
| 7 | 41 | 41 | 39 | 80 | 71 | 89 | 96 | 94 | 96 |
| 8 | 44 | 43 | 38 | 78 | 81 | 89 | 95 | 96 | 96 |
| 9 | 42 | 41 | 34 | 81 | 80 | 87 | 96 | 96 | 96 |
| 10 | 38 | 40 | 37 | 77 | 85 | 98 | 96 | 96 | 97 |
| 11 | 42 | 41 | 33 | 85 | 86 | 100 | 96 | 96 | 97 |
| 12 | 48 | 40 | 35 | 78 | 91 | 92 | 95 | 97 | 96 |
| 13 | 42 | 39 | 36 | 83 | 85 | 91 | 96 | 97 | 96 |
| 14 | 41 | 41 | 30 | 84 | 91 | 105 | 96 | 97 | 97 |
| 15 | 42 | 41 | 38 | 86 | 89 | 80 | 97 | 97 | 93 |
| 16 | 35 | 34 | 34 | 96 | 93 | 89 | 97 | 97 | 96 |
| 17 | 40 | 40 | 38 | 90 | 95 | 100 | 97 | 97 | 97 |
| 18 | 36 | 36 | 31 | 93 | 93 | 92 | 97 | 97 | 94 |
| *pre-ing = pre ingestion; post-ing = post ingestion; post-ex = 2 minutes post exercise;*  *pCO_2_ = partial pressure of carbon dioxide; pO_2_ = partial pressure of oxygen; SO_2_ = oxygen saturation* | | | | | | | | | |

**exhaustive graded exercise (placebo)**

| **n** | **IAT**  [km•h^-1^] | **V_max_**  [km•h^-1^] | **VO_2peak_**  [ml•min^-1^•kg^-1^l] | **BLa rest**  [mmol•l^-1^] | **BLa max**  [mmol•l^-1^] | **HR max**  [bpm] |
| --- | --- | --- | --- | --- | --- | --- |
| 1 | 13.0 | 17.1 | 60.4 | 0.77 | 15.50 | 199 |
| 2 | 14.0 | 16.2 | 70.3 | 0.50 | 5.96 | 188 |
| 3 | 13.7 | 17.1 | 62.4 | 0.68 | 7.18 | 184 |
| 4 | 13.3 | 16.5 | 59.8 | 0.74 | 7.92 | 197 |
| 5 | 14.4 | 18.0 | 63.9 | 0.60 | 8.20 | 188 |
| 6 | 13.8 | 17.1 | 57.0 | 0.50 | 7.20 | 186 |
| 7 | 13.1 | 16.2 | 54.2 | 1.24 | 9.56 | 186 |
| 8 | 13.9 | 16.2 | 57.9 | 0.50 | 8.69 | 186 |
| 9 | 12.6 | 16.2 | 61.7 | 0.58 | 8.16 | 189 |
| 10 | 14.0 | 17.4 | 59.2 | 0.66 | 6.01 | 182 |
| 11 | 15.8 | 18.9 | 64.9 | 0.72 | 10.30 | 176 |
| 12 | 15.5 | 18.8 | 58.0 | 0.85 | 8.40 | 194 |
| 13 | 13.3 | 16.2 | 52.0 | 0.57 | 7.48 | 190 |
| 14 | 14.6 | 18.6 | 68.4 | 0.55 | 7.24 | 181 |
| 15 | 13.5 | 18.0 | 74.1 | 0.96 | 8.65 | 184 |
| 16 | 12.8 | 16.2 | 53.8 | 0.51 | 9.21 | 201 |
| 17 | 13.4 | 17.1 | 70.0 | 1.16 | 10.5 | 192 |
| 18 | 12.5 | 16.2 | 53.1 | 0.67 | 6.65 | 186 |
| *IAT= individual anaerobic threshold; V_max_= maximum running speed; BLa rest= blood lactate concentration at rest;*  *BLa max= maximum blood lactate concentration; HR max = maximum heart rate* | | | | | | |

**exhaustive graded exercise (BICA)**

| **n** | **pH pre-ing** | **pH post-ing** | **pH post-ex** | **SBi pre-ing**  [mmol•l^-1^] | **SBi post-ing**  [mmol•l^-1^] | **SBi post-ex**  [mmol•l^-1^] | **BE pre-ing**  [mmol•l^-1^] | **BE post-ing**  [mmol•l^-1^] | **BE post-ex**  [mmol•l^-1^] |
| --- | --- | --- | --- | --- | --- | --- | --- | --- | --- |
| 1 | 7.40 | 7.48 | 7.17 | 28 | 35 | 13 | 4 | 11 | -14 |
| 2 | 7.42 | 7.48 | 7.42 | 25 | 31 | 21 | 1 | 7 | -2 |
| 3 | 7.41 | 7.47 | 7.35 | 25 | 30 | 19 | 1 | 6 | -6 |
| 4 | 7.41 | 7.47 | 7.36 | 27 | 35 | 24 | 3 | 11 | -1 |
| 5 | 7.41 | 7.47 | 7.25 | 26 | 31 | 15 | 2 | 7 | -11 |
| 6 | 7.41 | 7.49 | 7.35 | 25 | 32 | 18 | 1 | 9 | -7 |
| 7 | 7.44 | 7.48 | 7.27 | 26 | 31 | 16 | 3 | 8 | -10 |
| 8 | 7.42 | 7.49 | 7.38 | 27 | 35 | 23 | 3 | 11 | -1 |
| 9 | 7.40 | 7.52 | 7.33 | 27 | 35 | 20 | 3 | 11 | -5 |
| 10 | 7.39 | 7.53 | 7.29 | 25 | 33 | 18 | 0 | 9 | -7 |
| 11 | 7.40 | 7.48 | 7.36 | 25 | 32 | 20 | 1 | 8 | -4 |
| 12 | 7.41 | 7.48 | 7.21 | 25 | 31 | 14 | 1 | 7 | -13 |
| 13 | 7.41 | 7.50 | 7.27 | 22 | 29 | 16 | 2 | 8 | -7 |
| 14 | 7.44 | 7.50 | 7.30 | 26 | 33 | 15 | 2 | 9 | -10 |
| 15 | 7.42 | 7.51 | 7.32 | 24 | 27 | 16 | 3 | 11 | -7 |
| 16 | 7.41 | 7.42 | 7.29 | 24 | 24 | 15 | 0 | 0 | -11 |
| 17 | 7.43 | 7.47 | 7.35 | 27 | 34 | 22 | 3 | 10 | -3 |
| 18 | 7.38 | 7.58 | 7.19 | 22 | 27 | 12 | -2 | 5 | -15 |
| *pre-ing = pre ingestion; post-ing = post ingestion; post-ex = 2 minutes post exercise;*  *SBi = sodium bicarbonate concentration; BE = base excess* | | | | | | | | | |

**exhaustive graded exercise (BICA)**

| **n** | **pCO_2_ pre-ing**  [mmHg] | **pCO_2_ post-ing**  [mmHg] | **pCO_2_ post-ex**  [mmHg] | **pO_2_ pre-ing**  [mmHg] | **pO_2_ post-ing**  [mmHg] | **pO_2_ post-ex**  [mmHg] | **SO_2_ pre-ing**  [%] | **SO_2_ post-ing**  [%] | **SO_2_ post-ex**  [%] |
| --- | --- | --- | --- | --- | --- | --- | --- | --- | --- |
| 1 | 47 | 48 | 36 | 80 | 77 | 87 | 95 | 96 | 94 |
| 2 | 40 | 42 | 33 | 91 | 84 | 101 | 97 | 97 | 98 |
| 3 | 41 | 42 | 36 | 78 | 89 | 91 | 95 | 97 | 97 |
| 4 | 43 | 48 | 44 | 89 | 71 | 83 | 97 | 95 | 96 |
| 5 | 42 | 43 | 36 | 76 | 79 | 85 | 95 | 96 | 95 |
| 6 | 39 | 43 | 33 | 78 | 86 | 93 | 95 | 97 | 97 |
| 7 | 39 | 42 | 36 | 88 | 90 | 82 | 97 | 97 | 95 |
| 8 | 42 | 46 | 40 | 80 | 76 | 80 | 96 | 96 | 95 |
| 9 | 45 | 43 | 38 | 75 | 81 | 78 | 95 | 97 | 95 |
| 10 | 42 | 39 | 39 | 75 | 71 | 89 | 95 | 95 | 96 |
| 11 | 41 | 44 | 37 | 87 | 110 | 100 | 97 | 98 | 97 |
| 12 | 40 | 42 | 35 | 73 | 71 | 91 | 95 | 95 | 95 |
| 13 | 42 | 43 | 36 | 83 | 81 | 83 | 96 | 96 | 95 |
| 14 | 39 | 42 | 32 | 91 | 82 | 103 | 97 | 97 | 97 |
| 15 | 43 | 45 | 37 | 75 | 85 | 92 | 95 | 97 | 96 |
| 16 | 38 | 38 | 32 | 89 | 88 | 99 | 97 | 97 | 97 |
| 17 | 42 | 47 | 42 | 93 | 87 | 74 | 97 | 97 | 94 |
| 18 | 39 | 29 | 32 | 78 | 138 | 88 | 95 | 99 | 95 |
| *pre-ing= pre ingestion; post-ing = post ingestion; post-ex = 2 minutes post exercise;*  *pCO_2_ = partial pressure of carbon dioxide; pO_2_ = partial pressure of oxygen; SO_2_ = oxygen saturation* | | | | | | | | | |

**exhaustive graded exercise (BICA)**

| **n** | **IAT**  [km•h^-1^] | **V_max_**  [km•h^-1^] | **VO_2peak_**  [ml•min^-1^•kg^-1^l] | **BLa rest**  [mmol•^-1^l] | **BLa max**  [mmol•^-1^l] | **HR max**  [bpm] |
| --- | --- | --- | --- | --- | --- | --- |
| 1 | 12.9 | 17.1 | 54.5 | 0.88 | 18.80 | 205 |
| 2 | 13.7 | 16.2 | 58.3 | 0.58 | 7.24 | 184 |
| 3 | 13.3 | 17.1 | 65.0 | 0.58 | 10.00 | 186 |
| 4 | 13.5 | 16.2 | 55.0 | 0.88 | 9.59 | 185 |
| 5 | 14.5 | 18.9 | 62.2 | 0.85 | 11.50 | 189 |
| 6 | 13.5 | 17.1 | 55.4 | 0.66 | 6.60 | 189 |
| 7 | 13.5 | 16.8 | 53.8 | 0.66 | 12.60 | 172 |
| 8 | 14.0 | 17.1 | 55.3 | 0.50 | 11.00 | 189 |
| 9 | 13.1 | 17.1 | 54.6 | 0.69 | 13.80 | 193 |
| 10 | 13.6 | 18.0 | 70.2 | 1.20 | 8.23 | 183 |
| 11 | 15.8 | 18.9 | 66.1 | 0.77 | 13.50 | 183 |
| 12 | 14.4 | 19.1 | 64.4 | 1.01 | 11.40 | 196 |
| 13 | 12.8 | 16.2 | 52.0 | 0.50 | 10.50 | 192 |
| 14 | 14.5 | 18.9 | 63.8 | 0.54 | 6.11 | 191 |
| 15 | 13.7 | 18.0 | 66.5 | 0.93 | 11.80 | 184 |
| 16 | 12.8 | 16.2 | 56.0 | 0.50 | 11.50 | 199 |
| 17 | 13.6 | 17.7 | 73.1 | 0.69 | 13.40 | 194 |
| 18 | 12.2 | 16.2 | 51.1 | 1.07 | 8.24 | 190 |
| *IAT= individual anaerobic threshold; V_max_= maximum running speed; BLa rest= blood lactate concentration at rest;*  *BLa max= maximum blodd lactate concentration; HR max = maximum heart rate* | | | | | | |

**constant load test (placebo)**

| **n** | **pH pre-ing** | **pH post-ing** | **pH warm-up** | **pH 5 min** | **pH 10 min** | **pH 15 min** | **pH 20 min** | **pH 25 min** | **pH 30 min** | **pH 35 min** | **pH 40 min** | **pH 45 min** | **pH 50 min** | **pH post-ex** |
| --- | --- | --- | --- | --- | --- | --- | --- | --- | --- | --- | --- | --- | --- | --- |
| 1 | 7.43 | 7.41 | 7.40 | 7.35 | 7.33 | 7.34 | 7.35 | 7.34 | 7.37 | 7.28 |  |  |  | 7.26 |
| 2 | 7.42 | 7.45 | 7.46 | 7.45 | 7.44 | 7.46 | 7.45 | 7.46 | 7.47 |  |  |  |  | 7.39 |
| 3 | 7.42 | 7.43 | 7.42 | 7.40 | 7.40 | 7.40 | 7.40 | 7.41 | 7.41 | 7.35 |  |  |  | 7.35 |
| 4 | 7.40 | 7.40 | 7.40 | 7.37 | 7.38 | 7.38 | 7.38 | 7.41 | 7.40 |  |  |  |  | 7.36 |
| 5 | 7.43 | 7.42 | 7.42 | 7.40 | 7.38 | 7.39 | 7.38 | 7.40 | 7.39 | 7.33 |  |  |  | 7.31 |
| 6 | 7.40 | 7.40 | 7.41 | 7.39 | 7.39 | 7.41 | 7.41 | 7.43 | 7.44 | 7.36 |  |  |  | 7.35 |
| 7 | 7.38 | 7.41 | 7.42 | 7.38 | 7.41 | 7.41 | 7.42 | 7.42 | 7.42 | 7.35 |  |  |  | 7.32 |
| 8 | 7.40 | 7.42 | 7.40 | 7.38 | 7.39 | 7.39 | 7.39 | 7.39 | 7.40 | 7.34 | 7.31 |  |  | 7.33 |
| 9 | 7.42 | 7.43 | 7.42 | 7.40 | 7.40 | 7.41 | 7.40 | 7.42 | 7.41 | 7.40 | 7.38 |  |  | 7.37 |
| 10 | 7.42 | 7.42 | 7.42 | 7.38 | - | 7.39 | 7.40 | 7.39 | 7.41 | 7.35 |  |  |  | 7.34 |
| 11 | 7.40 | 7.42 | 7.41 | 7.39 | 7.40 | 7.41 | 7.41 | 7.42 | 7.42 |  |  |  |  | 7.35 |
| 12 | 7.39 | 7.42 | 7.40 | 7.36 | 7.35 | 7.36 | 7.37 | 7.34 | 7.36 |  |  |  |  | 7.32 |
| 13 | 7.39 | 7.38 | 7.37 | 7.34 | 7.36 | 7.37 | 7.38 | 7.37 | 7.37 |  |  |  |  | 7.31 |
| 14 | 7.44 | 7.43 | 7.42 | 7.39 | 7.41 | 7.42 | 7.40 | 7.42 | 7.41 | 7.36 | 7.37 | 7.37 | 7.37 | 7.38 |
| 15 | 7.41 | 7.43 | 7.41 | 7.43 | 7.43 | 7.44 | 7.45 | 7.44 | 7.45 | 7.40 |  |  |  | 7.37 |
| 16 | 7.39 | 7.40 | 7.41 | 7.39 | 7.38 | 7.37 | 7.42 | 7.40 | 7.41 | 7.38 |  |  |  | 7.38 |
| 17 | 7.39 | 7.43 | 7.44 | 7.41 | 7.42 | 7.41 | 7.41 | 7.42 | 7.42 | 7.39 | 7.39 | 7.37 |  | 7.39 |
| 18 | 7.42 | 7.42 | 7.42 | 7.38 | 7.37 | 7.37 | 7.36 | 7.36 | 7.36 | 7.26 | 7.19 |  |  | 7.18 |
| *pre = pre ingestion; post = post ingestion; warm-up = post warm-up. post ex = 2 minutes post exercise* | | | | | | | | | | | | | | |

**constant load test (placebo)**

| **n** | **SBi**  **pre-ing**  [mmol•l^-1^] | **SBi**  **post-ing**  [mmol•l^-1^] | **SBi**  **warm-up**  [mmol•l^-1^] | **SBi**  **5 min**  [mmol•l^-1^] | **SBi**  **10 min**  [mmol•l^-1^] | **SBi**  **15 min**  [mmol•l^-1^] | **SBi**  **20 min**  [mmol•l^-1^] | **SBi**  **25 min**  [mmol•l^-1^] | **SBi**  **30 min**  [mmol•l^-1^] | **SBi**  **35 min**  [mmol•l^-1^] | **SBi**  **40 min**  [mmol•l^-1^] | **SBi**  **45 min**  [mmol•l^-1^] | **SBi**  **50 min**  [mmol•l^-1^] | **SBi**  **post-ex**  [mmol•l^-1^] |
| --- | --- | --- | --- | --- | --- | --- | --- | --- | --- | --- | --- | --- | --- | --- |
| 1 | 27 | 26 | 24 | 22 | 21 | 21 | 21 | 19 | 20 | 15 |  |  |  | 14 |
| 2 | 25 | 24 | 25 | 25 | 25 | 24 | 24 | 25 | 24 |  |  |  |  | 20 |
| 3 | 26 | 26 | 25 | 23 | 23 | 23 | 23 | 23 | 24 | 20 |  |  |  | 19 |
| 4 | 26 | 25 | - | 25 | 24 | 23 | 24 | 24 | 24 |  |  |  |  | 21 |
| 5 | 25 | 26 | 25 | 22 | 21 | 22 | 22 | 22 | 22 | 18 |  |  |  | 16 |
| 6 | 24 | 23 | 24 | 22 | 22 | 22 | 21 | 22 | 22 | 17 |  |  |  | 16 |
| 7 | 25 | 26 | 25 | 22 | 24 | 23 | 24 | 24 | 24 | 20 |  |  |  | 16 |
| 8 | 27 | 27 | 26 | 25 | 25 | 20 | 25 | 24 | 25 | 21 | 19 |  |  | 18 |
| 9 | 25 | 25 | 25 | 23 | 23 | 23 | 23 | 23 | 21 | 20 | 19 |  |  | 18 |
| 10 | 24 | 25 | 24 | 23 | 25 | 24 | 24 | 24 | 25 | 22 |  |  |  | 19 |
| 11 | 25 | 25 | 25 | 24 | 24 | 24 | 23 | 24 | 23 |  |  |  |  | 19 |
| 12 | 25 | 25 | 23 | 21 | 21 | 21 | 21 | 20 | 21 |  |  |  |  | 18 |
| 13 | 23 | 21 | 21 | 19 | 19 | 19 | 19 | 19 | 18 |  |  |  |  | 15 |
| 14 | 26 | 25 | 24 | 23 | 24 | 23 | 24 | 25 | 24 | 21 | 19 | 19 | 19 | 18 |
| 15 | 25 | 27 | 26 | 24 | 24 | 24 | 24 | 25 | 25 | 21 |  |  |  | 21 |
| 16 | 23 | 23 | 21 | 23 | 22 | 21 | 23 | 22 | 22 | 20 |  |  |  | 20 |
| 17 | 25 | 27 | 28 | 26 | 26 | 26 | 26 | 26 | 26 | 24 | 23 | 21 |  | 22 |
| 18 | 23 | 22 | 22 | 20 | 19 | 18 | 18 | 17 | 17 | 13 | 10 |  |  | 9 |
| *pre-ing = pre ingestion; post -ing = post ingestion; warm-up = post warm-up. post-ex = 2 minutes post exercise; SBi = sodium bicarbonate concentration* | | | | | | | | | | | | | | |

**constant load test (placebo)**

| **n** | **BE**  **post-ing**  [mmol•l^-1^] | **BE**  **30 min**  [mmol•l^-1^] | **BE**  **post-ex**  [mmol•l^-1^] | **pCO_2_**  **post-ing**  [mmHg] | **pCO_2_**  **30 min**  [mmHg] | **pCO_2_**  **post-ex**  [mmHg] | **pO_2_**  **post-ing**  [mmHg] | **pO_2_**  **30 min**  [mmHg] | **pO_2_**  **post-ex**  [mmHg] | **SO_2_**  **post-ing**  [%] | **SO_2_**  **30 min**  [%] | **SO_2_**  **post-ex**  [%] |
| --- | --- | --- | --- | --- | --- | --- | --- | --- | --- | --- | --- | --- |
| 1 | 3 | -4 | -12 | 43 | 36 | 31 | 85 | 79 | 88 | 96 | 95 | 95 |
| 2 | 1 | 1 | -4 | 36 | 33 | 33 | 99 | 89 | 95 | 98 | 97 | 97 |
| 3 | 2 | 0 | -6 | 40 | 38 | 35 | 90 | 72 | 82 | 98 | 94 | 95 |
| 4 | 1 | 0 | -3 | 42 | 40 | 39 | 86 | 69 | 74 | 97 | 94 | 94 |
| 5 | 2 | -2 | -9 | 41 | 36 | 34 | 85 | 69 | 74 | 96 | 94 | 94 |
| 6 | -1 | -1 | -9 | 38 | 34 | 29 | 97 | 83 | 92 | 98 | 96 | 97 |
| 7 | 2 | 0 | -9 | 42 | 38 | 33 | 86 | 63 | 76 | 96 | 92 | 94 |
| 8 | 3 | 1 | -7 | 43 | 41 | 35 | 80 | 67 | 75 | 96 | 93 | 94 |
| 9 | 1 | -2 | -6 | 38 | 34 | 32 | 89 | 67 | 75 | 97 | 93 | 95 |
| 10 | 1 | 1 | -6 | 39 | 40 | 37 | 102 | 79 | 81 | 96 | 95 | 95 |
| 11 | 1 | -1 | -6 | 39 | 35 | 35 | 105 | 88 | 92 | 98 | 97 | 97 |
| 12 | 1 | -3 | -7 | 39 | 39 | 37 | 92 | 72 | 83 | 97 | 94 | 95 |
| 13 | -3 | -6 | -10 | 37 | 32 | 31 | 99 | 84 | 92 | 97 | 96 | 96 |
| 14 | 1 | 0 | -6 | 39 | 38 | 32 | 96 | 87 | 97 | 98 | 97 | 97 |
| 15 | 3 | 1 | -4 | 42 | 36 | 36 | 87 | 76 | 67 | 97 | 95 | 93 |
| 16 | -1 | -5 | -5 | 38 | 36 | 34 | 92 | 81 | 89 | 97 | 97 | 97 |
| 17 | 3 | 2 | -3 | 41 | 42 | 36 | 89 | 67 | 76 | 97 | 93 | 95 |
| 18 | -2 | -7 | -18 | 35 | 31 | 25 | 98 | 77 | 94 | 97 | 95 | 96 |
| *post-ing = post ingestion; post-ex = 2 minutes post exercise;*  *BE = base excess; pCO_2_ = partial pressure of carbon dioxide; pO_2_ = partial pressure of oxygen; SO_2_ = oxygen saturation* | | | | | | | | | | | | |

**constant load test (placebo)**

| **n** | **BLa**  **rest**  [mmol•l^-1^]] | **BLa**  **warm-up**  [mmol•l^-1^] | **BLa**  **5 min**  [mmol•l^-1^] | **BLa**  **10 min**  [mmol•l^-1^] | **BLa**  **15 min**  [mmol•l^-1^] | **BLa**  **20 min**  [mmol•l^-1^] | **BLa**  **25 min**  [mmol•l^-1^] | **BLa**  **30 min**  [mmol•l^-1^] | **BLa**  **35 min**  [mmol•l^-1^] | **BLa**  **40 min**  [mmol•l^-1^] | **BLa**  **45 min**  [mmol•l^-1^] | **BLa**  **50 min**  [mmol•l^-1^] | **BLa**  **top**  [mmol•l^-1^] |
| --- | --- | --- | --- | --- | --- | --- | --- | --- | --- | --- | --- | --- | --- |
| 1 | 0.96 | 1.31 | 4.19 | 4.91 | 5.09 | 5.12 | 5.67 | 6.19 | 10.60 |  |  |  | 11.60 |
| 2 | 0.74 | 0.50 | 1.21 | 1.01 | 1.19 | 1.32 | 1.22 | 1.24 | 4.92 |  |  |  | 4.92 |
| 3 | 0.75 | 0.82 | 1.97 | 2.07 | 2.17 | 2.22 | 2.10 | 2.17 | 4.87 |  |  |  | 5.74 |
| 4 | 0.80 | 0.54 | 1.61 | 1.39 | 1.30 | 1.42 | 1.44 | 1.51 | 3.50 |  |  |  | 3.50 |
| 5 | 0.65 | 0.86 | 2.26 | 2.28 | 2.33 | 2.40 | 2.58 | 3.05 | 5.09 |  |  |  | 7.13 |
| 6 | 0.89 | 0.81 | 2.53 | 2.45 | 2.61 | 3.06 | 3.04 | 2.63 | 6.50 | 7.67 |  |  | 7.67 |
| 7 | 0.72 | 0.63 | 2.17 | 1.76 | 1.76 | 1.84 | 2.00 | 2.06 | 5.26 | 7.77 |  |  | 7.77 |
| 8 | 0.65 | 1.24 | 2.88 | 2.85 | 2.51 | 2.37 | 2.64 | 2.44 | 5.35 | 7.05 |  |  | 6.77 |
| 9 | 0.50 | 0.66 | 1.94 | 2.01 | 2.29 | 2.46 | 2.45 | 2.52 | 4.03 | 5.65 |  |  | 5.72 |
| 10 | 0.78 | 0.81 | 1.87 | 1.37 | 1.24 | 1.19 | 1.12 | 1.21 | 3.55 | 4.29 |  |  | 4.29 |
| 11 | 0.52 | 0.51 | 1.92 | 1.78 | 1.63 | 1.71 | 1.66 | 1.88 | 5.49 |  |  |  | 5.49 |
| 12 | 0.56 | 0.98 | 3.40 | 3.29 | 2.61 | 3.02 | 2.70 | 3.19 |  |  |  |  | 5.29 |
| 13 | 0.50 | 1.00 | 3.36 | 3.41 | 3.40 | 3.47 | 3.39 | 3.95 |  |  |  |  | 6.40 |
| 14 | 0.50 | 0.56 | 1.88 | 1.68 | 1.63 | 1.81 | 1.44 | 1.51 | 4.37 | 5.61 | 5.91 | 6.07 | 6.03 |
| 15 | 0.78 | 1.60 | 2.88 | 2.38 | 2.38 | 2.41 | 2.58 | 2.50 | 4.23 | 5.40 |  |  | 5.40 |
| 16 | 0.87 | 0.79 | 2.61 | 2.40 | 2.44 | 2.24 | 2.30 | 2.27 | 4.95 |  |  |  | 4.19 |
| 17 | 0.60 | 0.50 | 1.53 | 1.61 | 1.68 | 1.59 | 1.84 | 1.72 | 3.13 | 4.28 | 5.54 | 5.68 | 5.68 |
| 18 | 0.63 | 0.93 | 3.33 | 3.52 | 4.29 | 4.59 | 4.70 | 4.95 | 8.35 | 11.10 |  |  | 12.40 |
| *BLa rest= blood lactate concentration at rest; BLa warm-up = blood lactate concentration after warm-up; BLa top= highest test specific blood lactate concentration* | | | | | | | | | | | | | |

**constant load test (placebo)**

| **n** | **HR 5 min**  [bpm] | **HR 10 min**  [bpm] | **HR 15 min**  [bpm] | **HR 20 min**  [bpm] | **HR 25 min**  [bpm] | **HR 30 min**  [bpm] | **HR 35 min**  [bpm] | **HR 40 min**  [bpm] | **HR 45 min**  [bpm] | **HR 50 min**  [bpm] | **HR top**  [bpm] |
| --- | --- | --- | --- | --- | --- | --- | --- | --- | --- | --- | --- |
| 1 | 176 | 183 | 188 | 189 | 191 | 194 | 200 |  |  |  | 203 |
| 2 | 155 | 160 | 162 | 164 | 169 | 171 | 186 |  |  |  | 186 |
| 3 | 165 | 171 | 174 | 175 | 176 | 177 | 185 |  |  |  | 186 |
| 4 | 158 | 160 | 163 | 167 | 168 | 170 | 185 |  |  |  | 185 |
| 5 | 170 | 173 | 176 | 177 | 178 | 180 | 185 |  |  |  | 188 |
| 6 | 145 | 151 | 152 | 157 | 158 | 164 | 181 | 187 |  |  | 187 |
| 7 | 150 | 152 | 154 | 156 | 157 | 161 | 170 | 172 |  |  | 172 |
| 8 | 162 | 170 | 167 | 170 | 174 | 177 | 187 | 191 |  |  | 188 |
| 9 | 163 | 167 | 169 | 170 | 171 | 175 | 186 | 189 |  |  | 190 |
| 10 | 153 | 158 | 159 | 160 | 161 | 164 | 178 | 183 |  |  | 183 |
| 11 | 154 | 161 | 160 | 163 | 166 | 167 | 176 |  |  |  | 176 |
| 12 | 171 | 177 | 178 | 180 | 182 | 185 |  |  |  |  | 192 |
| 13 | 165 | 170 | 174 | 175 | 180 | 182 |  |  |  |  | 190 |
| 14 | 159 | 163 | 164 | 165 | 165 | 167 | 175 | 178 | 179 | 180 | 182 |
| 15 | 158 | 160 | 161 | 162 | 163 | 165 | 176 | 178 |  |  | 178 |
| 16 | 174 | 178 | 179 | 180 | 183 | 185 | 191 |  |  |  | 187 |
| 17 | 153 | 156 | 160 | 161 | 162 | 164 | 179 | 183 | 185 | 187 | 187 |
| 18 | 163 | 168 | 172 | 173 | - | - | 182 | 184 |  |  |  |
| *HR top = highest test specific heart rate* | | | | | | | | | | | |

**constant load test (placebo)**

| **n** | **TTE**  [min] | **VO_2_ top**  [l•min^-1^l] | **VCO_2_ top**  [l•min^-1^l] | **VE top**  [l•min^-1^l] |
| --- | --- | --- | --- | --- |
| 1 | 37.5 | 4.62 | 4.46 | 151.0 |
| 2 | 35.0 | 4.78 | 5.02 | 151.6 |
| 3 | 38.5 | 4.93 | 4.58 | 107.8 |
| 4 | 35.0 | 4.67 | 4.78 | 110.3 |
| 5 | 39.0 | 4.50 | 4.37 | 151.1 |
| 6 | 40.0 | 4.15 | 4.61 | 166.3 |
| 7 | 40.0 | 4.12 | 4.09 | 107.4 |
| 8 | 42.5 | 4.01 | 3.95 | 129.9 |
| 9 | 43.0 | 3.39 | 3.09 | - |
| 10 | 40.0 | 4.68 | 4.19 | 105.9 |
| 11 | 35.0 | 4.05 | 4.65 | 121.6 |
| 12 | 33.5 | 3.55 | 4.20 | 104.3 |
| 13 | 34.0 | 4.21 | 4.47 | 155.6 |
| 14 | 54.0 | 3.62 | 4.21 | 123.4 |
| 15 | 40.0 | 3.69 | 4.05 | 100.1 |
| 16 | 33.0 | 1.68 | 1.71 | 104.7 |
| 17 | 50.0 | 4.03 | 4.07 | 110.8 |
| 18 | 42.5 | 2.72 | 2.75 | 124.4 |
| *TTE = time to exhaustion; VO_2_ top= highest test specific oxygen consumption*  *VCO_2_ top= highest test specific carbon dioxide output; VE top= highest test specific ventilation* | | | | |

**constant load test (BICA)**

| **n** | **pH pre-ing** | **pH post-ing** | **pH warm-up** | **pH 5 min** | **pH 10 min** | **pH 15 min** | **pH 20 min** | **pH 25 min** | **pH 30 min** | **pH 35 min** | **pH 40 min** | **pH 45 min** | **pH 50 min** | **pH post-ex** |
| --- | --- | --- | --- | --- | --- | --- | --- | --- | --- | --- | --- | --- | --- | --- |
| 1 | 7.43 | 7.49 | 7.44 | 7.38 | 7.39 | 7.39 | 7.39 | 7.39 | 7.40 | 7.35 |  |  |  | 7.27 |
| 2 | 7.42 | 7.48 | 7.48 | 7.47 | 7.48 | 7.48 | 7.48 | 7.48 | 7.49 |  |  |  |  | 7.38 |
| 3 | 7.40 | 7.48 | 7.48 | 7.45 | 7.45 | 7.46 | 7.48 | 7.47 | 7.48 |  |  |  |  | 7.44 |
| 4 | 7.41 | 7.44 | 7.46 | 7.45 | 7.46 | 7.46 | 7.46 | 7.48 | 7.50 | 7.46 |  |  |  | 7.50 |
| 5 | 7.43 | 7.47 | 7.47 | 7.47 | 7.43 | 7.44 | 7.45 | 7.44 | 7.45 | 7.39 | 7.35 |  |  | 7.35 |
| 6 | 7.43 | 7.49 | 7.48 | 7.48 | 7.49 | 7.48 | 7.46 | 7.48 | 7.49 |  |  |  |  | 7.46 |
| 7 | 7.41 | 7.51 | 7.49 | 7.47 | 7.48 | 7.49 | 7.49 | 7.48 | 7.50 | 7.38 |  |  |  | 7.43 |
| 8 | 7.38 | 7.49 | 7.46 | 7.44 | 7.45 | 7.45 | 7.47 | 7.47 | 7.46 | 7.42 |  |  |  | 7.41 |
| 9 | 7.43 | 7.50 | 7.44 | 7.45 | 7.47 | 7.46 | 7.48 | 7.49 | 7.48 |  |  |  |  | 7.48 |
| 10 | 7.43 | 7.49 | 7.49 | 7.45 | 7.47 | 7.47 | 7.47 | 7.46 | 7.48 | 7.43 | 7.41 |  |  | 7.39 |
| 11 | 7.41 | 7.48 | 7.46 | 7.45 | 7.46 | 7.48 | 7.47 | 7.47 | 7.47 | 7.41 |  |  |  | 7.43 |
| 12 | 7.41 | 7.47 | 7.49 | 7.44 | 7.45 | 7.45 | 7.45 | 7.45 | 7.45 |  |  |  |  | 7.42 |
| 13 | 7.40 | 7.45 | 7.47 | 7.44 | 7.44 | 7.43 | 7.45 | 7.45 | 7.45 |  |  |  |  | 7.44 |
| 14 | 7.43 | 7.51 | 7.46 | 7.44 | 7.46 | 7.45 | 7.45 | 7.47 | 7.48 | 7.43 | 7.42 | 7.42 | 7.40 | 7.41 |
| 15 | 7.41 | 7.51 | 7.47 | 7.47 | 7.46 | 7.48 | 7.48 | 7.48 | 7.48 | 7.46 |  |  |  | 7.44 |
| 16 | 7.41 | 7.42 | 7.43 | 7.40 | 7.40 | 7.42 | 7.43 | 7.43 | 7.44 |  |  |  |  | 7.40 |
| 17 | 7.41 | 7.48 | 7.48 | 7.46 | 7.44 | 7.47 | 7.46 | 7.44 | 7.46 | 7.43 |  |  |  | 7.42 |
| 18 | 7.40 | 7.49 | 7.45 | 7.45 | 7.44 | 7.43 | 7.43 | 7.44 | 7.40 | 7.30 |  |  |  | 7.21 |
| *pre-ing = pre ingestion; post-ing = post ingestion; warm-up = post warm-up. post-ex = 2 minutes post exercise* | | | | | | | | | | | | | | |

**constant load test (BICA)**

| **n** | **SBi**  **pre-ing**  [mmol•l^-1^] | **SBi**  **post-ing**  [mmol•l^-1^] | **SBi**  **warm-up**  [mmol•l^-1^] | **SBi**  **5 min**  [mmol•l^-1^] | **SBi**  **10 min**  [mmol•l^-1^] | **SBi**  **15 min**  [mmol•l^-1^] | **SBi**  **20 min**  [mmol•l^-1^] | **SBi**  **25 min**  [mmol•l^-1^] | **SBi**  **30 min**  [mmol•l^-1^] | **SBi**  **35 min**  [mmol•l^-1^] | **SBi**  **40 min**  [mmol•l^-1^] | **SBi**  **45 min**  [mmol•l^-1^] | **SBi**  **50 min**  [mmol•l^-1^] | **SBi**  **post-ex**  [mmol•l^-1^] |
| --- | --- | --- | --- | --- | --- | --- | --- | --- | --- | --- | --- | --- | --- | --- |
| 1 | 30 | 35 | 33 | 29 | 27 | 26 | 25 | 24 | 25 | 20 |  |  |  | 15 |
| 2 | 24 | 28 | 30 | 28 | 26 | 26 | 26 | 26 | 26 |  |  |  |  | 19 |
| 3 | 23 | 30 | 30 | 29 | 28 | 28 | 28 | 28 | 29 |  |  |  |  | 24 |
| 4 | 28 | 32 | 30 | 29 | 30 | 31 | 31 | 30 | 31 | 26 |  |  |  | 26 |
| 5 | 26 | 31 | 31 | 30 | 28 | 28 | 27 | 28 | 28 | 24 | 21 |  |  | 19 |
| 6 | 25 | 31 | 31 | 26 | 27 | 28 | 26 | 26 | 26 |  |  |  |  | 23 |
| 7 | 25 | 32 | 33 | 30 | 29 | 29 | 30 | 29 | 29 | 18 |  |  |  | 25 |
| 8 | 24 | 32 | 32 | 29 | 29 | 29 | 30 | 29 | 29 | 24 |  |  |  | 24 |
| 9 | 27 | 32 | 30 | 29 | 29 | 29 | 28 | 28 | 28 |  |  |  |  | 26 |
| 10 | 25 | 33 | 33 | 31 | 31 | 31 | 30 | 31 | 31 | 27 | 24 |  |  | 22 |
| 11 | 26 | 31 | 30 | 30 | 29 | 29 | 27 | 28 | 29 | 22 |  |  |  | 22 |
| 12 | 24 | 31 | 32 | 27 | 30 | 29 | 28 | 28 | 28 |  |  |  |  | 23 |
| 13 | 24 | 30 | 29 | 26 | 25 | 25 | 25 | 25 | 23 |  |  |  |  | 22 |
| 14 | 26 | 31 | 31 | 29 | 28 | 30 | 29 | 29 | 29 | 26 | 23 | 23 | 22 | 22 |
| 15 | 26 | 32 | 32 | 28 | 29 | 30 | 31 | 31 | 29 | 28 | 26 |  |  | 26 |
| 16 | 23 | 25 | 26 | 21 | 24 | 24 | 24 | 24 | 24 |  |  |  |  | 21 |
| 17 | 27 | 34 | 34 | 31 | 31 | 32 | 31 | 30 | 31 | 29 |  |  |  | 23 |
| 18 | 24 | 30 | 30 | 27 | 24 | 22 | 23 | 22 | 20 | 16 |  |  |  | 12 |
| *pre-ing = pre ingestion; post -ing = post ingestion; warm-up = post warm-up. post-ex = 2 minutes post exercise; SBi = sodium bicarbonate concentration* | | | | | | | | | | | | | | |

**constant load test (BICA)**

| **n** | **BE**  **post-ing**  [mmol•l^-1^] | **BE**  **30 min**  [mmol•l^-1^] | **BE**  **post-ex**  [mmol•l^-1^] | **pCO_2_**  **post-ing**  [mmHg] | **pCO_2_**  **30 min**  [mmHg] | **pCO_2_**  **post-ex**  [mmHg] | **pO_2_**  **post-ing**  [mmHg] | **pO_2_**  **30 min**  [mmHg] | **pO_2_**  **post-ex**  [mmHg] | **SO_2_**  **post-ing**  [%] | **SO_2_**  **30 min**  [%] | **SO_2_**  **post-ex**  [%] |
| --- | --- | --- | --- | --- | --- | --- | --- | --- | --- | --- | --- | --- |
| 1 | 11 | 1 | -10 | 46 | 41 | 34 | 82 | 71 | 80 | 95 | 94 | 94 |
| 2 | 4 | 3 | -5 | 38 | 35 | 33 | 94 | 76 | 98 | 98 | 96 | 97 |
| 3 | 6 | 5 | 1 | 40 | 39 | 36 | 87 | 73 | 75 | 97 | 95 | 95 |
| 4 | 8 | 7 | 3 | 47 | 40 | 34 | 80 | 65 | 73 | 96 | 94 | 96 |
| 5 | 7 | 4 | -6 | 42 | 41 | 35 | 80 | 60 | 119 | 96 | 91 | 98 |
| 6 | 7 | 3 | -1 | 40 | 34 | 32 | 85 | 74 | 78 | 97 | 96 | 96 |
| 7 | 8 | 6 | 1 | 41 | 37 | 38 | 76 | 56 | 64 | 96 | 91 | 93 |
| 8 | 8 | 6 | 0 | 42 | 42 | 39 | 79 | 57 | 70 | 96 | 91 | 94 |
| 9 | 8 | 5 | 3 | 42 | 39 | 36 | 85 | 74 | 85 | 97 | 95 | 97 |
| 10 | 9 | 7 | -2 | 43 | 42 | 37 | 66 | 80 | 82 | 94 | 95 | 96 |
| 11 | 7 | 5 | -2 | 42 | 40 | 33 | 93 | 78 | 79 | 97 | 96 | 96 |
| 12 | 7 | 4 | -1 | 43 | 40 | 36 | 93 | 75 | 82 | 97 | 95 | 96 |
| 13 | 6 | -1 | -2 | 44 | 34 | 33 | 88 | 74 | 83 | 97 | 95 | 96 |
| 14 | 8 | 5 | -2 | 40 | 39 | 36 | 100 | 100 | 81 | 98 | 98 | 96 |
| 15 | 8 | 6 | 2 | 40 | 40 | 37 | 94 | 70 | 66 | 98 | 95 | 94 |
| 16 | 1 | 0 | -3 | 39 | 36 | 35 | 98 | 76 | 83 | 98 | 95 | 96 |
| 17 | 10 | 7 | -1 | 45 | 43 | 37 | 80 | 66 | 69 | 96 | 93 | 94 |
| 18 | 6 | -4 | -14 | 40 | 33 | 32 | 92 | 81 | 89 | 97 | 96 | 95 |
| *post-ing = post ingestion; post-ex = 2 minutes post exercise;*  *BE = base excess; pCO_2_ = partial pressure of carbon dioxide; pO_2_ = partial pressure of oxygen; SO_2_ = oxygen saturation* | | | | | | | | | | | | |

**constant load test (BICA)**

| **n** | **BLa**  **rest**  [mmol•l^-1^]] | **BLa**  **warm-up**  [mmol•l^-1^] | **BLa**  **5 min**  [mmol•l^-1^] | **BLa**  **10 min**  [mmol•l^-1^] | **BLa**  **15 min**  [mmol•l^-1^] | **BLa**  **20 min**  [mmol•l^-1^] | **BLa**  **25 min**  [mmol•l^-1^] | **BLa**  **30 min**  [mmol•l^-1^] | **BLa**  **35 min**  [mmol•l^-1^] | **BLa**  **40 min**  [mmol•l^-1^] | **BLa**  **45 min**  [mmol•l^-1^] | **BLa**  **50 min**  [mmol•l^-1^] | **BLa**  **top**  [mmol•l^-1^] |
| --- | --- | --- | --- | --- | --- | --- | --- | --- | --- | --- | --- | --- | --- |
| 1 | 0.91 | 2.75 | 6.75 | 8.36 | 8.58 | 8.63 | 8.33 | 8.64 | 12.60 | 16.90 |  |  | 16.90 |
| 2 | 0.50 | 0.50 | 2.00 | 2.14 | 2.57 | 2.91 | 3.04 | 2.92 | 7.98 |  |  |  | 7.98 |
| 3 | 0.92 | 1.00 | 2.31 | 2.46 | 2.72 | 2.52 | 2.70 | 2.76 | 5.86 |  |  |  | 5.86 |
| 4 | 0.65 | 0.61 | 1.91 | 1.69 | 1.72 | 1.83 | 1.84 | 1.94 | 4.51 |  |  |  | 4.97 |
| 5 | 0.61 | 0.97 | 2.31 | 2.39 | 2.47 | 2.50 | 2.88 | 2.85 | 6.09 | 8.64 |  |  | 8.98 |
| 6 | 0.96 | 1.14 | 3.22 | 3.01 | 3.43 | 3.70 | 3.79 | 3.55 | 7.31 |  |  |  | 7.31 |
| 7 | 0.89 | 1.14 | 2.97 | 2.59 | 2.50 | 2.59 | 2.59 | 2.72 | 5.83 | 8.46 |  |  | 8.46 |
| 8 | 0.50 | 1.25 | 2.55 | 2.75 | 2.47 | 2.46 | 3.05 | 2.66 | 5.78 | 6.77 |  |  | 6.77 |
| 9 | 0.66 | 0.93 | 2.64 | 2.70 | 2.71 | 2.99 | 3.00 | 2.69 |  |  |  |  | 4.40 |
| 10 | 0.75 | 0.87 | 1.88 | 1.70 | 1.67 | 1.73 | 1.82 | 1.72 | 4.70 | 6.04 | 6.78 |  | 6.78 |
| 11 | 0.90 | 0.95 | 2.55 | 2.46 | 2.74 | 3.39 | 3.57 | 3.37 | 7.51 |  |  |  | 8.14 |
| 12 | 0.50 | 0.56 | 2.81 | 2.50 | 2.37 | 2.26 | 2.48 | 2.60 |  |  |  |  | 4.71 |
| 13 | 1.35 | 1.69 | 4.23 | 4.58 | 4.66 | 4.67 | 5.12 | 5.65 |  |  |  |  | 6.15 |
| 14 | 0.61 | 0.65 | 2.70 | 2.27 | 2.44 | 2.46 | 2.36 | 2.31 | 5.41 | 7.02 | 7.53 | 8.65 | 8.44 |
| 15 | 0.59 | 1.37 | 2.88 | 2.57 | 2.38 | 2.40 | 2.41 | 2.36 | 4.17 | 5.33 | 5.8 |  | 5.80 |
| 16 | 0.86 | 1.01 | 2.76 | 2.68 | 2.52 | 2.62 | 2.64 | 2.76 |  |  |  |  | 3.84 |
| 17 | 0.60 | 0.60 | 2.16 | 2.06 | 2.45 | 2.57 | 2.62 | 2.55 | 4.88 | 6.38 | 8.48 |  | 8.48 |
| 18 | 1.09 | 2.15 | 4.23 | 4.83 | 5.63 | 6.06 | 6.80 | 7.58 | 12.50 | 16.00 |  |  | 16.00 |
| *BLa rest= blood lactate concentration at rest; BLa warm-up = blood lactate concentration after warm-up; BLa top= highest test specific blood lactate concentration* | | | | | | | | | | | | | |

**constant load test (BICA)**

| **n** | **HR 5 min**  [bpm] | **HR 10 min**  [bpm] | **HR 15 min**  [bpm] | **HR 20 min**  [bpm] | **HR 25 min**  [bpm] | **HR 30 min**  [bpm] | **HR 35 min**  [bpm] | **HR 40 min**  [bpm] | **HR 45 min**  [bpm] | **HR 50 min**  [bpm] | **HR top**  [bpm] |
| --- | --- | --- | --- | --- | --- | --- | --- | --- | --- | --- | --- |
| 1 | 158 | 164 | 166 | 170 | 173 | 174 | 189 |  |  |  | 189 |
| 2 | 166 | 168 | 168 | 169 | 169 | 171 | 181 |  |  |  | 181 |
| 3 | 166 | 166 | 168 | 171 | 172 | 172 | 185 |  |  |  | 182 |
| 4 | 168 | 171 | 174 | 175 | 178 | 179 | 185 | 188 |  |  | 187 |
| 5 | 155 | 162 | 161 | 163 | 169 | 167 | 185 |  |  |  | 185 |
| 6 | 153 | 155 | 156 | 159 | 159 | 162 | 172 | 173 |  |  | 173 |
| 7 | 168 | 173 | 174 | 175 | 177 | 178 | 187 | 191 |  |  | 191 |
| 8 | 158 | 163 | 163 | 168 | 169 | 172 |  |  |  |  | 180 |
| 9 | 158 | 160 | 161 | 164 | 164 | 168 | 180 | 184 | 186 |  | 186 |
| 10 | 158 | 162 | 162 | 166 | 167 | 170 | 181 |  |  |  | 178 |
| 11 | 174 | 178 | 182 | 183 | 186 | 188 |  |  |  |  | 195 |
| 12 | 168 | 172 | 176 | 178 | 182 | 184 |  |  |  |  | 185 |
| 13 | 158 | 159 | 162 | 163 | 165 | 166 | 174 | 177 | 178 | 177 | 178 |
| 14 | 153 | 156 | 158 | 160 | 161 | 161 | 173 | 176 | 177 |  | 177 |
| 15 | 176 | 179 | 184 | 184 | 187 | 189 |  |  |  |  | 192 |
| 16 | 158 | 163 | 164 | 168 | 170 | 171 | 184 | 189 | 190 |  | 190 |
| 17 | 167 | 174 | 177 | 179 | 177 | 178 | 180 |  |  |  |  |
| 18 | 158 | 164 | 166 | 170 | 173 | 174 | 189 |  |  |  | 189 |
| *HR top = highest test specific heart rate* | | | | | | | | | | | |

**constant load test (BICA)**

| **n** | **TTE**  [min] | **VO_2_ top**  [l•min^-1^l] | **VCO_2_ top**  [l•min^-1^l] | **VE top**  [l•min^-1^l] |
| --- | --- | --- | --- | --- |
| 1 | 40.0 | 4.70 | 4.95 | 152.2 |
| 2 | 35.0 | 4.96 | 5.10 | 161.2 |
| 3 | 35.0 | 4.54 | 4.31 | 104.4 |
| 4 | 37.0 | 4.58 | 3.80 | 99.7 |
| 5 | 42.5 | 4.46 | 4.31 | 155.5 |
| 6 | 35.0 | 4.37 | 4.90 | 183.5 |
| 7 | 40.0 | 3.16 | 3.56 | 92.1 |
| 8 | 40.0 | 4.32 | 4.44 | 130.2 |
| 9 | 34.0 | 3.41 | 3.47 | 106.6 |
| 10 | 45.0 | 4.43 | 4.17 | 110.2 |
| 11 | 37.0 | 4.62 | 4.12 | 127.8 |
| 12 | 33.5 | 3.60 | 3.34 | 104.8 |
| 13 | 32.0 | 4.20 | 4.30 | 133.2 |
| 14 | 54.5 | 4.36 | 4.25 | 100.7 |
| 15 | 45.0 | 3.84 | 3.90 | 98.5 |
| 16 | 37.5 | 3.17 | 3.29 | 109.1 |
| 17 | 45.0 | 3.41 | 4.17 | 114.1 |
| 18 | 40.0 | 2.69 | 3.09 | 122.7 |
| *TTE = time to exhaustion; VO_2_ top= highest test specific oxygen consumption*  *VCO_2_ top= highest test specific carbon dioxide output; VE top= highest test specific ventilation* | | | | |
